# Prioritizing drug targets by perturbing biological network response functions

**DOI:** 10.1371/journal.pcbi.1012195

**Published:** 2024-06-27

**Authors:** Matthew C. Perrone, Michael G. Lerner, Matthew Dunworth, Andrew J. Ewald, Joel S. Bader

**Affiliations:** 1 Institute for Computational Medicine and Department of Biomedical Engineering, Johns Hopkins University, Baltimore, Maryland, United States of America; 2 Department of Physics, Engineering and Astronomy, Earlham College, Richmond, Indiana, United States of America; 3 Department of Cell Biology, Johns Hopkins University School of Medicine, Baltimore, Maryland, United States of America; 4 Department of Oncology, Sidney Kimmel Comprehensive Cancer Center, Baltimore, Maryland, United States of America; 5 Giovanis Institute for Translational Cell Biology, Johns Hopkins University School of Medicine, Baltimore, Maryland, United States of America; University at Buffalo - The State University of New York, UNITED STATES

## Abstract

Therapeutic interventions are designed to perturb the function of a biological system. However, there are many types of proteins that cannot be targeted with conventional small molecule drugs. Accordingly, many identified gene-regulatory drivers and downstream effectors are currently undruggable. Drivers and effectors are often connected by druggable signaling and regulatory intermediates. Methods to identify druggable intermediates therefore have general value in expanding the set of targets available for hypothesis-driven validation. Here we identify and prioritize potential druggable intermediates by developing a network perturbation theory, termed NetPert, for response functions of biological networks. Dynamics are defined by a network structure in which vertices represent genes and proteins, and edges represent gene-regulatory interactions and protein-protein interactions. Perturbation theory for network dynamics prioritizes targets that interfere with signaling from driver to response genes. Applications to organoid models for metastatic breast cancer demonstrate the ability of this mathematical framework to identify and prioritize druggable intermediates. While the short-time limit of the perturbation theory resembles betweenness centrality, NetPert is superior in generating target rankings that correlate with previous wet-lab assays and are more robust to incomplete or noisy network data. NetPert also performs better than a related graph diffusion approach. Wet-lab assays demonstrate that drugs for targets identified by NetPert, including targets that are not themselves differentially expressed, are active in suppressing additional metastatic phenotypes.

## Author summary

Many therapies, especially small molecule drugs, inhibit the activity of a protein target. Not all proteins are druggable, however. When the proteins known to be involved in a disease process are not druggable, identifying a good target is difficult. In cancer, for example, the somatic driver mutations are often known, and the downstream changes in gene expression may be measured, but the driver gene and response genes may not be druggable. Here we present a method, NetPert, that selects candidate drug targets based on their ability to perturb interactions connecting a driver gene to response genes. Drugs already known to target these candidates are also reported. Applications to mouse models for metastatic breast cancer show that targets ranked highly by this method are highly active in wet-lab assays for dissemination and metastatic outgrowth. The NetPert method provides an interpretable data-driven approach to target selection and drug prioritization.

## Introduction

Disease processes often involve upstream causal genes that initiate and regulate downstream effectors. In the context of cancer, the upstream genes are often called cancer drivers and are identified by somatic mutations and gene amplifications. For complex genetic disorders, genome-wide association studies may identify germ-line variants that similarly identify nearby causal genes. Downstream effectors are often identified by RNA sequencing.

When the practical goal is to block the disease process, a reasonable approach is to perturb the activity of the driver or response genes, often with a small molecule therapeutic. This approach can work well when the corresponding gene products belong to protein families that are readily targeted by drugs. However, known drivers and effectors are often not targetable. The important problem is then to identify additional targets that function in the pathway but may have been missed by the experimental analysis. Analysis of differential gene expression by RNA sequencing, for example, can miss signal transduction targets that regulate effectors but are not themselves differentially expressed. Furthermore, if the number of potential targets exceeds the throughput of an experimental assay, methods to prioritize candidate targets have additional value.

Nominating and prioritizing candidate targets is therefore an important problem. In cancer, drivers and effectors may come from observational studies involving tumor-versus-normal comparisons. Comparisons may also involve cancer cells at different stages of metastasis. Experimental model systems, including genetically engineered mouse models (GEMMs) and patient-derived xenograft models (PDXs), can provide direct routes to test drivers and validate perturbations [1–3]. These studies are enabled in part by organotypic cell culture, in which cells grown in 3D give access to phenotypes that replicate many important properties of tissues and organs [4–6].

Although 3D cell culture is far less expensive than animal models, assay throughput is still limited compared with traditional cancer cell line screens. A typical approach is to limit consideration to genetic drivers and differentially expressed genes that are targets of known cancer therapeutics. Connections between targets and existing drugs and chemical probes are available from resources such as the Drug Repurposing Hub [7], which cross-references protein targets with United States Food and Drug Administration (FDA) approved drugs, clinical trial drugs, and pre-clinical compounds. However, the number of druggable candidates in this intersection may be small: of the 125 canonical cancer mutational driver genes [8], only 54 are oncogenes, and only 37 of these are known drug targets.

**Fig 1:**
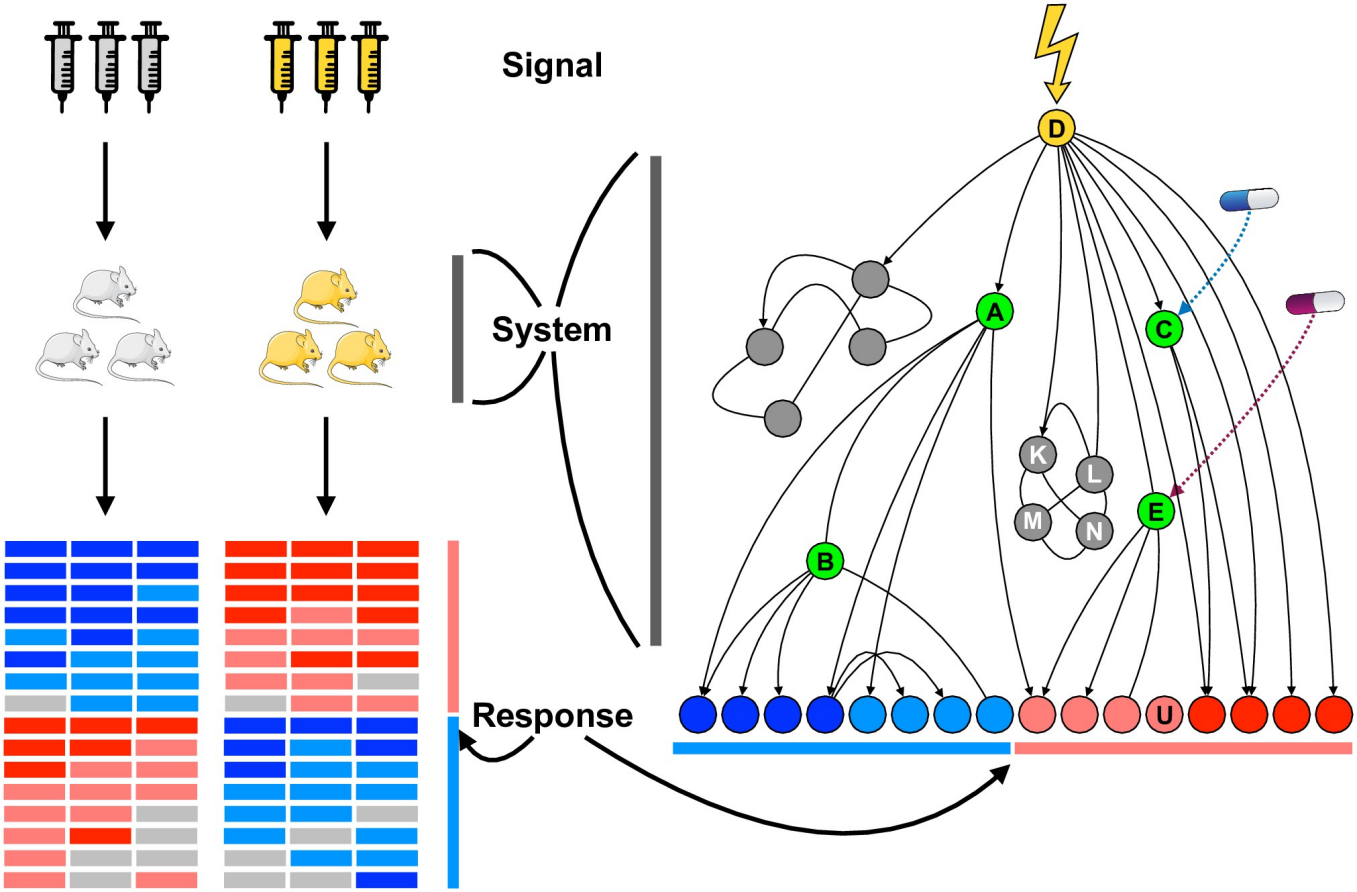
NetPert overview. The NetPert method represents an experiment as an input signal (top) and output response (bottom) governed by a system response function (middle). **Real-world experiment, left:** chemical or genetic inputs pair a control treatment (grey syringes) with one or more experimental drivers (yellow syringes). The system, depicted as mouse biological replicates, may comprise cell lines, organoids, or whole animals. Responses are often measured as differential gene expression of the experimental signal relative to the control, here represented as a heatmap, one column for each biological replicate, and rows clustered showing genes that are significantly up-regulated or down-regulated by the signal. **Computational network model, right:** The system is represented by genes and proteins (circles) connected by pairwise protein-protein interactions (line segments) and gene-regulatory interactions (arrows). The driver gene (labeled ‘D’ in yellow) corresponds to the known target of the signal, and the response genes at the bottom correspond to the up-regulated (red circles) and down-regulated genes (blue circles) from the experiment. Drugs can perturb the signaling response. **Genes A, B:** Gene A is directly attached to the driver and to a response gene, a category named DIR for driver-intermediate-response; gene B is on a path with two intermediates and is termed DIIR. **Genes C, E:** Gene C is not on any shortest paths from driver to response genes. Betweenness centrality ignores genes that are not on shortest paths, but NetPert can rank them highly. If genes C and E are targeted by known drugs, depicted as pills, NetPert suggests drug repurposing candidates. **Genes K, L, M, N:** While highly connected clusters often generate high rankings in network-based methods, NetPert only considers the KLMN cluster to the extent that it contributes to the response function of regulated genes. **Gene U:** This response gene is unconnected in the network model, possibly due to incompleteness of interaction databases and forms of regulation not yet incorporated into the network model.

The approach we describe, NetPert, uses perturbation theory to identify intermediates that may be valuable targets even though they are not themselves cancer drivers or differentially expressed effectors (Fig 1). Our computational model includes genes and proteins as components. Dynamics are defined by protein-protein interactions, gene-regulatory interactions, as well as degradation. We use a linear systems approach to define the response function between driver and response genes. We then use perturbation theory to define the importance of intermediate genes to the response.

We show that the short-time limit of the perturbation theory leads to an importance measure that is similar to betweenness centrality (BC) [9, 10], but without the limitation of requiring genes to be on shortest paths from driver to response. Our method performs better than betweenness centrality, primarily from the ability to rank genes that are not on shortest paths. For unit interaction strengths, the network model is similar to graph diffusion or network propagation methods, which we have used previously to identify candidate genes interacting with genes of interest [11–13] and which have been broadly useful in analyzing biological networks [14–18]. Our expression derived from perturbation theory gives better performance than a previous formulation.

To demonstrate the value of NetPert, we apply the method to an experimental model for metastatic breast cancer involving directed activation of *Twist1* [1], whose gene product is a transcription factor regulator of the epithelial-mesenchymal transition in cancer [19, 20]. Expression of TWIST1 protein leads to robust cell dissemination in organoids. This same system was used to assay the ability of chemical and genetic perturbations to stop dissemination, with results reported for several differentially expressed druggable targets [21]. New experimental data presented here consider assays for colony formation, a model for outgrowth of micrometastatic lesions into macroscopic tumors. Targets for both the dissemination and the colonization stages of metastasis are needed in the clinic because metastasis is the major driver of mortality across cancer sites [22], yet treatment options remain limited. To validate NetPert, we show that the experimental effect of perturbing targets correlates more strongly with rankings from NetPert than with rankings from differential expression, betweenness centrality, and a graph diffusion-based method, TieDIE [23]. Better performance is observed across multiple protein interaction databases [24–26] combined with gene-regulatory interactions [27]. NetPert provides new capabilities for identifying and prioritizing targetable intermediates that interfere with pathways connecting drivers and effectors of disease-related phenotypes.

## Materials and methods

### Biological network model

A biological network is represented as a dynamical system with vertex *i* representing a gene and its protein product, and *x*_*i*_ representing its activity. Activity here is taken generally as transcript count, protein abundance, or protein activity subject to post-translational modifications. Although the actual dynamics of a cell are highly non-linear, we assume a near-equilibrium setting in which linear response theory is an appropriate approximation. In this limit, we define *a*_*ij*_ as the kinetic rate constant for activation or repression of gene or protein *i* by *j*, and *d*_*i*_ as a degradation or loss term due to RNase or protease activity, or possibly due to dilution upon cell division. These dynamics define a set of ordinary differential equations,

$$\begin{array}{c} \frac{d}{dt}x_{i}\left(t\right)=a_{ij}x_{j}\left(t\right)-d_{i}x_{i}\left(t\right). \end{array}$$ (1)

These equations are summarized in matrix form with activation matrix **A** whose terms are *a*_*ij*_ and decay matrix **D** whose terms are *d*_*ij*_ = *d*_*i*_*δ*_*ij*_, where *δ*_*ij*_ is the Kronecker (discrete) *δ*-function, 1 for *i* = *j* and 0 otherwise. With **x** representing the vector of gene or protein activities,

$$\begin{array}{c} \frac{d}{dt}x\left(t\right)=Ax\left(t\right)-Dx\left(t\right)\equiv Hx\left(t\right), \end{array}$$

which defines the time evolution operator **H** = **A** − **D**.

The dynamics of the system are then fully determined by the matrix exponential of the time evolution operator,

$$\begin{array}{c} x\left(t+t_{0}\right)=\text{exp}\left(Ht\right)x\left(t_{0}\right)\equiv G\left(t\right)x\left(t_{0}\right), \end{array}$$

which in turn defines the two-vertex Green’s function **G**(*t*) with terms *g*_*ij*_(*t*) = [exp(**H***t*)]_*ij*_. In a time-independent system, with no explicit time dependence in **H**, it is convenient to define the origin of time *t*_0_ as 0.

The time response function for the network is defined as the change in activity of gene *i* due to a change in gene *j* at a previous time, with time 0 specified by convention,

$$\begin{array}{c} \frac{dx_{i}\left(t\right)}{dx_{j}\left(0\right)}=\frac{d}{dx_{j}\left(0\right)}\sum_{k}\;g_{ik}\left(t\right)x_{k}\left(0\right)=g_{ij}\left(t\right), \end{array}$$

because *dx*_*k*_(0)/*dx*_*j*_(0) = *δ*_*jk*_. The time response function is therefore the Green’s function. According to the fluctuation-dissipation theorem [28–31], this response function could in principle be measured as an equilibrium time correlation function.

### First-order perturbation theory

Perturbations often take the form of enhancements to activity by over-expression or reductions to activity by gene knockdown or small molecule inhibition. The perturbed dynamics are

$$\begin{array}{c} H_{\Lambda }=H+\Lambda , \end{array}$$

where the perturbation matrix **Λ** is diagonal with terms λ_*kl*_ = λ_*k*_*δ*_*kl*_. The perturbed Green’s function is

$$\begin{array}{c} G_{\Lambda }\left(t\right)=\text{exp}\left[Ht+\Lambda t\right]. \end{array}$$

The matrices **H** and **Λ** do not commute, and hence the perturbed Green’s function is not simply the product of matrix exponentials exp[**H***t*] exp[**Λ***t*]. The unperturbed system has **Λ** = 0, with **H**_0_ = **H** and **G**_0_ = **G**. This nomenclature defines a perturbation as a change in the time evolution operator rather than as a change in the system preparation, which is instead defined by the initial state **x**(0).

For perturbations close to equilibrium, the Green’s function can be approximated as

$$\begin{array}{c} G_{\Lambda }\left(t\right)=G_{\Lambda }{\left(t\right)|}_{\Lambda =0}+\sum_{k}\;\lambda_{k}\left(d/d\lambda_{k}\right)G_{\Lambda }\left(t\right){|}_{\Lambda =0}+{O(|\Lambda |}^{2}), \end{array}$$

with the derivatives evaluated in the reference system and O indicating asymptotic order. The derivatives of the response function term are often described as sensitivities,

$$\begin{array}{ccc} S_{k}\left(t\right) & \equiv & \frac{d}{d\lambda_{k}}G_{\Lambda }{\left(t\right)|}_{\Lambda =0}\\ s_{k;ij}\left(t\right) & = & {\left[S_{k}\left(t\right)\right]}_{ij}. \end{array}$$

The sensitivity, essentially the derivative of a two-vertex response function yielding a three-vertex function, is thus equivalent to a first-order perturbation theory for the response function. Sensitivity analysis has been effective in a wide range of chemical kinetics and related problems [32].

In our context, sensitivity is valuable in permitting calculations of a perturbed system based on the properties of the unperturbed reference system. Alternative perturbations could be envisioned; for example, a component could be removed entirely. Removal is less likely to represent increased degradation by shRNA knockdown or partial inhibition by a small molecule; it could be an improved representation for a gene knockout.

The sensitivity may be calculated using a path integral formulation [31, 33, 34]. The propagation time is discretized into *P* intervals,

$$\begin{array}{c} \text{exp}\left(H_{\Lambda }t\right)={\left[\text{exp}(H_{\Lambda }t/P)\right]}^{P}=\underset{P\to \infty }{\text{lim}}\;{\left[I+\frac{H_{\Lambda }t}{P}\right]}^{P}, \end{array}$$

with **I** denoting the identity matrix. Then, noting that (*d*/*d*λ_*k*_)**H**_Λ_ = **1**_*kk*_, a projection operator for vertex *k* expressed as a matrix with a 1 at row and column *k* and 0 elsewhere,

$$\begin{array}{ccc} \frac{d}{d\lambda_{k}}e^{H_{\Lambda }t} & = & \underset{P\to \infty }{\text{lim}}\;\sum_{p=1}^{P}\;{\left[I+\frac{H_{\Lambda }t}{P}\right]}^{P-p}\frac{1_{kk}t}{P}{\left[I+\frac{H_{\Lambda }t}{P}\right]}^{p-1}\\ & = & \underset{P\to \infty }{\text{lim}}\;\sum_{p=1}^{P}\;\frac{t}{P}\;\text{exp}\;\left[H_{\Lambda }\frac{(P-p)t}{P}\right]1_{kk}\;\text{exp}\;\left[H_{\Lambda }\frac{(p-1)t}{P}\right]. \end{array}$$

Defining *t*′ = *pt*/*P* and converting from a sum to an integral,

$$\begin{array}{c} \left(d/d\lambda_{k}\right)\;\text{exp}\;\left(H_{\Lambda }t\right)=\int_{0}^{t}\;dt^{\prime }\;\text{exp}\left[H_{\Lambda }\left(t-t^{\prime }\right)\right]1_{kk}\;\text{exp}\;\left[H_{\Lambda }t^{\prime }\right]. \end{array}$$

For the sensitivity, the matrix exponentials are evaluated with **Λ** = 0 and become Green’s functions for the unperturbed system. The sensitivity matrix for an intermediate vertex is therefore

$$\begin{array}{c} S_{k}\left(t\right)=\int_{0}^{t}\;dt^{\prime }\;G\left(t-t^{\prime }\right)1_{kk}G\left(t^{\prime }\right). \end{array}$$

The matrix elements are the sensitivities,

$$\begin{array}{c} s_{k;ij}\left(t\right)=\int_{0}^{t}\;dt^{\prime }\;g_{ik}\left(t-t^{\prime }\right)g_{kj}\left(t^{\prime }\right). \end{array}$$

If sets of driver genes, denoted *D*, and response genes, denoted *R*, are specified, we define the weight *w*_*k*_(*t*) of intermediate vertex *k* for the response at time *t* to be

$$\begin{array}{c} w_{k}\left(t\right)=\sum_{i\in R}\sum_{j\in D}\;s_{k;ij}\left(t\right). \end{array}$$ (2)

Weights calculated according to Eq 2 are termed NetPert-Endpoints because the importance of a driver or response gene on its own path is included. These strong self-terms bias the rankings to push driver and response genes to the top. To eliminate this bias, the final version of NetPert excludes self-terms:

$$\begin{array}{c} w_{k}\left(t\right)=\sum_{i\in R,i\neq k}\sum_{j\in D,j\neq k}s_{k;ij}\left(t\right). \end{array}$$ (3)

Unless specifically noted, results were generated using the final NetPert method, Eq 3, rather than the method including endpoint weights, NetPert-Endpoints, Eq 2. We used arithmetic spacing with *n*_*t*_ time intervals, each of length *t*/*n*_*t*_, to calculate the convolutions. The propagator **G**(*t*/*n*_*t*_) was calculated within numerical error using the Scipy matrix exponential function, and then self-multiplied to obtain propagator values at the required time points to perform the convolution integral.

### Graph diffusion isomorphism and parameter selection

Often the kinetic parameters in the linear model, Eq 1, are unknown. A useful approach is to assign an equal nominal value to each reaction rate *a*_*ij*_, and then to apply a conservation of activity rule by setting the decay rate of a vertex to its outgoing activation rate,

$$\begin{array}{ccc} a_{ij} & = & 1\;\text{if}\;j\to i,\;0\;\text{if}\;\text{not}\;\text{connected};\\ d_{j} & = & \sum_{i}\;a_{ij}. \end{array}$$

Note that this model is not necessarily symmetric: for a unidirectional interaction from *j* to *i*, *a*_*ij*_ = 1 but *a*_*ji*_ = 0. With this model, *x*_*i*_(*t*) is interpreted as the probability that a random walker is located at vertex *i* at time *t* for a continuous-time random walk. The time evolution operator **H** is the negative of the graph Laplacian **L** ≡ **D** − **A**, and the Green’s function element *g*_*ij*_(*t*) is the conditional probability that a random walker is located at vertex *i* at time *t* given that it was at vertex *j* at time 0.

These dynamics are unnormalized in the sense that the rate that a random walker leaves a vertex is not uniform but instead is proportional to the vertex degree. Normalized dynamics would have time evolution operator

$$\begin{array}{c} H_{\text{normalized}}=AD^{-1}-I, \end{array}$$

where **I** is the identity matrix and **D** is the diagonal out-degree matrix as before. With normalized dynamics, the rate of activation of *i* by *j* is 1/*d*_*j*_ rather than 1.

We prefer the unnormalized form for two reasons. First, reaction rates in reality are more likely to be dependent on the type of biochemical reaction than the number of targets. Thus, kinases are likely to have similar reaction rates regardless of the number of targets.

Second, the main observable in the dynamic model is the vertex density, which usually randomizes to approach a right eigenvector with eigenvalue 0. For unnormalized dynamics with a symmetric transition matrix, **A** = **A**^**⊺**^, corresponding to a random walk on an undirected graph, (**A** − **D**) ⋅ **1** = **0**, and the vector **1** with each entry 1 is an eigenvector with eigenvalue 0. If the graph is connected, all the other eigenvalues of **A** − **D** are negative, and vertices reach equal density exponentially quickly [35]. For normalized dynamics, however, the vector of vertex degrees has eigenvalue 0, and the equilibrium distribution has much higher density for high-degree vertices. Statistics based on random walk probabilities are then highly biased in favor of high-degree vertices, particularly for biological networks with a long-tailed degree distribution.

Returning to the unnormalized dynamics, the loss of density from an initial state provides an implicit scaling of time. Consider a system prepared with *x*_*j*_(0) = *δ*_*ji*_, with vertex *i* the only driver. The Green’s function element *g*_*ii*_(*t*) defines the activity of vertex *i* after time *t*. Note that the Green’s function sums over paths of all lengths and includes contributions from paths that have departed *i* and then returned any number of times. For relaxation parameter *r* ∈ [0, 1], we define relaxation time *τ*_*r*_ as

$$\begin{array}{c} \tau_{r}\equiv {\text{arg}\;\text{min}}_{t}\;t:g_{ii}\left(t\right)=1-r. \end{array}$$

In other words, *τ*_*r*_ is the first passage time at which a fraction *r* of the density has escaped from the driver vertex to the rest of the network.

Let *D* denote the set of drivers (not to be confused with the degree matrix **D**), and let |*D*| denote the number of drivers. We prepare the system at time 0 with each driver having equal starting density 1/|*D*|. The time *τ*_*r*_ is defined as

$$\begin{array}{c} \tau_{r}\equiv {\text{arg}\;\text{min}}_{t}\;t:\frac{1}{\left|D\right|}[\sum_{i\in D,j\in D}g_{ij}\left(t\right)]=1-r. \end{array}$$

At time *t* = 0, all the density is at the drivers, and *τ*_0_ = 0. In the short-time limit for a single driver *i* with out-degree *d*_*i*_, *g*_*ii*_(*t*) ≈ exp(−*d*_*i*_*t*). This expression remains valid for longer times under a non-returning approximation, which is appropriate for large networks where density becomes randomized over all vertices. Under the non-returning approximation,

$$\tau_{r}\approx -\frac{1}{d_{i}}\;\text{ln}\;\left(1-r\right).$$

We used the *τ*_*r*_ calculated from this approximation for simulations and confirmed that the density remaining at the driver was close to the requested value of *r* (see Robustness to the response time and convolution time-step). Unless noted otherwise, results correspond to half the density remaining at the drivers, *r* = 1/2, and *τ*_*r*_ ≈ (ln 2)/*d*_*i*_.

### Betweenness centrality implementation

Betweenness centrality (BC) was computed using betweenness_centrality_subset from the Python network analysis package NetworkX [36], which implements a standard BC algorithm to consider paths between a subset of sources and targets [37]. The algorithm input consisted of a NetworkX directed graph with the same nodes and unweighted edges as the NetPert network, the driver as the source, and the differentially expressed response genes as the target subset. The algorithm returned a dictionary of nodes with betweenness centrality as the value.

### Graph diffusion implementation

We re-implemented the Tied Diffusion through Interacting Events (TieDIE) method [23] in Python. The original TieDIE method tested three approaches for the relevance function: HotNet [38], which was introduced using an undirected heat diffusion process for protein-protein interactions; topic-sensitive or personalized PageRank [39], which incorporates directed edges in a random walk; and signaling pathway impact analysis (SPIA) [40], which incorporates both the directionality and regulatory mode of an interaction. For the comparison with NetPert, we used the HotNet approach with a heat diffusion process that was undirected for protein-protein interactions and directed for gene-regulatory interactions. This approach was chosen for two reasons. First, HotNet was the method preferred by TieDIE because it was computationally efficient and performed comparably with personalized PageRank at identifying true network paths in the presence of false-positive interactions over moderate levels of recall. Second, undirected protein-protein interactions and directed gene-regulatory interactions permitted us to compare TieDIE to NetPert using the same network topology and diffusion kernel. The HotNet relevance function for propagation time *t*′ is

$$\begin{array}{c} v\left[x\left(t_{0}\right),A\right]=\text{exp}\;\left[\left(A-D\right)t^{\prime }\right]x\left(t_{0}\right), \end{array}$$

where **A** is the directed, but unsigned, version of the adjacency matrix with entries *a*_*ij*_ = 1 if there is a directed edge from gene *j* to *i* and 0 otherwise, **D** is a diagonal matrix with diagonal elements *d*_*jj*_ = ∑_*i*_ *a*_*ij*_ and off-diagonal elements *d*_*ij*_ = 0 for *i* ≠ *j*, and **x**(*t*_0_) is the vector of initial scores for diffusion. Thus, the modified HotNet relevance function is the matrix exponential of the time evolution operator in the unperturbed NetPert system.

In the TieDIE method, a vector of initial scores is given for each input set of genes. For comparison with NetPert, there were two input sets: the source set consisted of only the driver gene and the target set consisted of the differentially expressed response genes. The vector of initial scores for diffusion from the driver was denoted **x**(*t*_0_) and the vector of initial scores for diffusion from the response genes was denoted **y**(*t*_0_). For the diffusion process from the driver, the driver *i* was given the initial score of *x*_*i*_(*t*_0_) = 1; the other genes in the network were initialized to zero, *x*_*j*_(*t*_0_) = 0 for *j* ≠ *i*. For the diffusion process from the set of response genes, denoted *R*, each response gene was given the initial score *y*_*r*_(*t*_0_) = 1/|*R*|, *r* ∈ *R*, and the rest of the genes were given an initial score *y*_*j*_(*t*_0_) = 0, *j* ∉ *R*.

The linking score for TieDIE is

$$\begin{array}{c} z=\text{min}\;(v[x(t_{0}),A],\;v[y\left(t_{0}\right),A^{⊺}]), \end{array}$$ (4)

where **A**^**⊺**^ is the transpose the adjacency matrix, which is used to direct the diffusion process from the response genes in the reverse direction. The min operator is used to weight genes by their scores in both diffusion processes. We associated the total diffusion time 2*t*′ for TieDIE with the response time *t* in NetPert.

For comparing to NetPert, which ranks all intermediate genes, we used the linking score for the TieDIE ranking. The original TieDIE method also used a threshold to select a subset of linking genes and a filter to select a subset of edges logically consistent with the source set, the target set, and the identified linking genes. We did not use the threshold or the filter because they do not affect the linking scores or the TieDIE gene ranking.

The TieDIE and NetPert methods have essentially two differences. First, while TieDIE uses the min function to generate a linking function, NetPert uses a product form that follows from perturbation theory. Second, again as defined by perturbation theory, NetPert is a full convolution over intermediate times for the perturbation, whereas TieDIE effectively models the perturbation as an impulse at *t*/2.

### Short-time expansion, betweenness centrality, and graph diffusion

A short-time expansion of the vertex sensitivity has a close relationship with both betweenness centrality (BC) and the TieDIE method. In the short-time limit,

$$G\left(t\right)=\text{exp}\left[\left(A-D\right)t\right]=I+\left(A-D\right)t+O\left(t^{2}\right),$$

where *O* indicates the order of the neglected terms. Substituting into Eq 3, using *D* to represent the set of driver genes and *R* to represent the set of response genes, yields

$$w_{k}\left(t\right)=\sum_{i\in R,i\neq k}\sum_{j\in D,j\neq k}\int_{0}^{t}\;dt^{\prime }\;{\left[I+\left(A-D\right)\left(t-t^{\prime }\right)+O\left\{{\left(t-t^{\prime }\right)}^{2}\right\}\right]}_{ik}{\left[I+\left(A-D\right)t^{\prime }+O\left(t^{\prime 2}\right)\right]}_{kj}.$$

Note, however, that because of the endpoint-excluding condition, the elements of the diagonal matrices are zero: **I**_*ik*_ = **I**_*kj*_ = 0, and similarly *d*_*ik*_ = *d*_*kj*_ = 0. We therefore have

$$\begin{array}{ccc} w_{k}\left(t\right) & = & \sum_{i\in R,i\neq k}\sum_{j\in D,j\neq k}a_{ik}a_{kj}\int_{0}^{t}\;dt^{\prime }\;\left(t-t^{\prime }\right)t^{\prime }+O\left[\left(t-t^{\prime }\right)t^{\prime 2}+{\left(t-t^{\prime }\right)}^{2}t^{\prime }\right]\\ & = & \left(t^{3}/6\right)\sum_{i\in R,i\neq k}\sum_{j\in D,j\neq k}a_{ik}a_{kj}+O\left(t^{4}\right). \end{array}$$

The product *a*_*ik*_*a*_*kj*_ counts how many endpoint-excluding paths of length 2 from *j* to *i* pass through vertex *k*, and these paths must be shortest paths because endpoint-excluding paths must be length 2 or greater. Although lim_*t*→0_ *w*_*k*_(*t*) = 0, weights may still be compared for infinitesimal *t*. In this limit, the rank-order of a vertex is determined by the number of length-2 paths from the driver genes to the response genes that pass through the vertex. While the above analysis assumes at least one vertex *k* that is attached to both a driver and response gene (or equivalently that at least one length-2 path exists), the same logic holds where the shortest path requires more than a single intermediate vertex.

In comparison, the BC formula for a subset of vertices is

$$b_{k}=\sum_{i\in R,i\neq k}\sum_{j\in D,j\neq k}\frac{\sigma (j,i|k)}{\sigma (j,i)}$$

where *σ*(*j*, *i*) is the number of shortest paths from vertex *j* to *i* and *σ*(*j*, *i*|*k*) is the number of those shortest paths passing through vertex *k*.

While the short-time limit of NetPert resembles BC, NetPert nevertheless calculates a weight for each vertex on at least one path from driver to response, regardless of path length. For a given driver and response pair, a weight of order *t*^3^ is given to a vertex that lies on a path of length 2; a weight of *t*^4^ is given to a vertex that lies on a path of length 3; and in general a weight of order *t*^*n*+1^ is given to a vertex that lies on a path of length *n*. The total weight of a vertex between a driver and a set of responses is the sum of weights for all paths. This makes it possible that a vertex on no paths of length 2 between driver and responses could be ranked higher than a vertex on at least one path of length 2.

The short-time expansion of the Green’s function for the TieDIE linking score, Eq 4, is

$$\begin{array}{c} z_{k}\left(t\right)=\text{min}(\sum_{j\in D}{\left[I+\left(A-D\right)t+O\left(t^{2}\right)\right]}_{kj}x_{j},\;\sum_{i\in R}{\left[I+\left(A^{⊺}-U\right)t+O\left(t^{2}\right)\right]}_{ki}\;y_{i}), \end{array}$$

where the degree matrix for the diffusion process from the response genes **U** has terms *u*_*ij*_ = *u*_*i*_*δ*_*ij*_ and *u*_*i*_ = ∑_*j*_ *a*_*ij*_. Once again, because of the endpoint-excluding condition, the elements of the diagonal matrices are zero, **I**_*ik*_ = **I**_*kj*_ = 0, and similarly *d*_*ik*_ = *d*_*kj*_ = *u*_*ik*_ = *u*_*kj*_ = 0. For the single-driver condition we set *x*_*j*_(*t*_0_) = 1 and for each response gene *i* in the set of response genes *R*, we set *y*_*i*_(*t*_0_) = 1/|*R*|, *i* ∈ *R*. Therefore, the short-time limit of the TieDIE is

$$\begin{array}{c} z_{k}\left(t\right)=\text{min}(a_{kj}t+O\left(t^{2}\right),{\left|R\right|}^{-1}\;\sum_{i\in R,i\neq k}a_{ik}t+O\left(t^{2}\right)). \end{array}$$

The short-time limit expansions of the NetPert weight and the TieDIE linking score are similar in practice for a single driver. An intermediate connected to the driver and to |*R*′| out of the |*R*| response genes will have short-time limit |*R*′|*t*^3^/6 for NetPert and |*R*′|*t*/|*R*| for TieDIE, directly proportional in this limit. Thus, intermediates directly connected to a driver and response genes should be ranked in the same order by NetPert and TieDIE in the short-time limit, but intermediates on shortest paths of length 3 or more may be ranked differently.

### Network data

Mouse protein-protein interactions (PPIs) and gene-regulatory interactions were assembled from public sources. PPIs were downloaded from version 2021–05 of Integrated Interactions Database (IID), a database of PPIs in different species [24]. This dataset contained 528,707 experimentally detected and orthologous mouse interactions. Protein self-interactions were removed. Protein symbols were mapped from UniProt ID to Mouse Genome Informatics (MGI) accession ID using an Oct. 9, 2023, download of UniProt databases [41]. The MGI accession ID was then mapped to the MGI symbol using the report “List of Mouse Genetic Markers” from an Oct. 9, 2023, download of the Mouse Genome Database (MGD) [42]. A total of 539,606 unique PPIs were identified.

Gene-regulatory interactions were downloaded from Transcriptional Regulatory Relationships Unraveled by Sentence-based Text mining (TRRUST) version 2, a manually curated database of human and mouse transcriptional regulatory interactions [27]. The dataset contained 6,490 transcription factor-target regulatory interactions of 827 mouse transcription factors. The mode of regulation was not incorporated in NetPert because 40% of the interactions were missing this information and many of the interactions are context dependent, with annotations of activating in some cell states and repressing in other cell states. The MGI mouse gene symbols as reported by TRRUST were used.

In general, PPIs were treated as undirected, which means that existence of the interaction *a*_*ij*_ implies the existence of the edge in the reverse direction, *a*_*ji*_. Both directions were assigned the value 1. Gene-regulatory interactions are not generally symmetric and were treated as directed, with value *a*_*ij*_ = 1 for transcription factor *j* regulating gene *i*. A gene and its corresponding protein product were represented as a single vertex.

### Alternative databases of protein-protein interactions

The PPI datasets “HI-union” and “Lit-BM” were downloaded on Dec. 6, 2023, from the Human Reference Interactome (HuRI) [26]. The HI-union dataset includes all published screening data from the Center for Cancer Systems Biology (CCSB), consisting of 9,094 proteins and 64,006 interactions. The most recent CCSB screening effort, named HI-III-20, tested pairwise combinations of human protein-coding genes using high throughput yeast two-hybrid screens, producing 52,569 PPIs involving 8,275 proteins. The Lit-BM dataset consists of 13,441 PPIs, involving 6,047 proteins, found in the literature. Each interaction in Lit-BM is supported by at least two pieces of experimental evidence. The HI-union and Lit-BM datasets were combined. Human protein symbols were mapped from Ensembl ID to HGNC ID using HGNC databases. The HGNC IDs were then mapped to the MGI symbol orthologs using the report “Human and Mouse Homology Classes with Sequence Information” downloaded on Oct. 9, 2023, from MGD [42]. This mapping assumes that protein interactions are conserved between human and mouse orthologs. Protein self-interactions were removed. A total of 85,302 unique mouse PPIs were identified.

The mouse physical subnetwork dataset was downloaded on Dec. 8, 2023, from the Search Tool for Recurring Instances of Neighbouring Genes (STRING) version 12.0 [25]. The dataset consists of PPIs collected from experimental databases, protein complex and pathway knowledgebases, and parsing full-text journal articles and summary texts from online catalogs. The physical subnetwork consists of 707,162 interactions involving 16,912 proteins. Mouse STRING protein IDs were mapped to MGI symbols using STRING databases. Protein self-interactions were removed. A total of 352,463 unique PPIs were identified.

Similar to the IID network, PPIs from both HuRI and STRING were treated as undirected with both directions assigned the same value *a*_*ij*_ = *a*_*ji*_ = 1 and a gene and its corresponding protein product were represented as a single vertex.

### Driver and response genes for epithelial cell dissemination

Driver and response genes were obtained from a mouse model of epithelial cell dissemination in breast cancer. The model used mouse mammary organoids with *Twist1* under an inducible promoter [1]. Expression of *Twist1* generated dissemination of epithelial cells from organoids cultured in Matrigel, a 3D extracellular matrix. We used the RNA sequencing analysis data from the sheet “All Sequenced Genes” of Table S1 of Ref. [1], providing normalized read counts, log 2-fold changes, z-scores, p-values, and multiple testing corrected p-values for 18,260 genes for *Twist1*-expressing versus control mouse organoid differential expression. The genes were identified by the MGI gene symbol along with corresponding Entrez ID, and Human Genome Organisation (HUGO) Gene Nomenclature Committee (HGNC) human ortholog symbol, if available. The 183 genes differentially expressed between *Twist1*-expressing and control mouse organoids at the genome-wide significance level of 2.7 × 10^−6^ for a 0.05 family-wise error rate were used as response genes in this study. The published study raw data, which we did not use, is available as Sequence Read Archive SRP033275.

### Drug repurposing data

The Drug Repurposing Hub [7] version from March 24, 2020, was used to identify small molecules and approved drugs suggested to have activity against protein targets. The Drug Repurposing Hub includes 4,382 small molecules targeting a total of 2,183 human proteins. Small molecules without protein targets were excluded from our analysis. Human proteins were mapped to mouse orthologs using the report “Human and Mouse Homology Classes with Sequence Information” from MGD [42]. This mapping assumes that drug activity is conserved between human and mouse orthologs. Small molecules identified in this way often have activity described against multiple targets, and biological activity can depend on interactions with one or more of these targets. Consequently, small molecules with more than five targets listed in the Drug Repurposing Hub were not included.

### Dissemination inhibition analysis

Pharmacological inhibition data from Figure 1b of Ref. [21] were accessed from https://github.com/EwaldLab/2019_Prkd1/. This dataset contained IC_50_ and dissemination scores normalized to vehicle control for small-molecule inhibitors and receptor antagonists of genes up-regulated by *Twist1*; inhibitors of matrix degradation, adhesion, and cell proliferation; clinically approved drugs; and inhibitors for the off-targets of inhibitors and receptor antagonists of genes up-regulated by *Twist1*. Protein targets were identified using information from the supplier, if available, or the Drug Repurposing Hub (S1 Table). Compounds often were described as having multiple potential targets, which makes interpretation more challenging without directed genetic perturbations. Therefore, compounds were assumed to inhibit potential targets equally and a target was assigned the most potent inhibition score if it was targeted by multiple tested compounds.

### Colony formation inhibition analysis

Tumors were isolated from the mouse mammary tumor virus-polyoma middle tumor-antigen (MMTV-PyMT) genetically engineered mouse model of breast cancer, as previously described [43]. Mechanical shaking, collagenase and trypsin enzymatic digestion, and differential centrifugations and filtering produced epithelial cell clusters of approximately two to ten cells. Clusters were robotically seeded into wells containing Matrigel, treated with organoid medium (+bFGF), and incubated overnight. Drugs and compounds were then dosed at 1*μ*mol/L into the wells and incubated for four days. After incubation, the samples were fixed with paraformaldehyde and imaged in 3D with a high-content analyzer. Resulting colonies were segmented from maximum intensity projections of each well using Fiji image processing software. Briefly, the background of each image was subtracted, the fluorescence signal was converted to binary, and colonies were separated using a watershed transformation. Colony counts and sizes were then output, and results were integrated with the applied compound name using Python. A colony formation score for each compound was calculated as the number of colonies formed in the presence of the compound as a percentage of the mean number of colonies formed in the presence of a vehicle control.

Drugs and compounds were sourced from the Approved Oncology Drugs (AOD) set IX library from the United States National Cancer Institute (NCI) Developmental Therapeutics Program (DTP) and the Epigenetic Compound (EC) library from MedChemExpress. The AOD library contains 147 FDA-approved oncology drugs. Drugs in the AOD library were screened in three biological replicates. The inhibition score was taken as the mean. The EC library contains 480 compounds targeting epigenetic processes, including some FDA-approved drugs. Protein targets were identified using the Drug Repurposing Hub. Of the 147 drugs in the AOD library, 124 had exact name matches with drugs in the Drug Repurposing Hub that had listed targets. The EC library had 190 of 480 compounds exactly match the names of compounds in the Drug Repurposing Hub with listed targets. Drugs and compounds were assumed to inhibit potential targets equally and a target was assigned the most potent inhibition score if it was targeted by multiple tested compounds. Assay results are provided separately for the AOD library (S2 Table) and the EC library (S3 Table).

### Gene and protein notation

The computational model represents a gene and its protein product as a combined single entity. The experimental datasets were generated from mouse models, and genes and proteins are therefore represented by mouse gene symbols. Conventionally, mouse gene symbols are italicized, with only the first letter uppercase, and protein symbols are in regular font, with all letters uppercase. Human gene symbols are italicized with all letters uppercase, and human protein symbols are regular font with all letters uppercase. Because human and mouse protein symbols are often identical for orthologs, the text makes clear when the distinction is important. Network views use regular font for readability.

### Implementation and availability

The NetPert method is implemented in Python with standard open-source libraries. The Graphviz library was used for graph drawing [44, 45]. The Fruchterman-Reingold force-directed placement algorithm [46] and the hierarchical placement algorithm dot [47] were used for graph layout.

Computation time for a MacOS 10.14.6 system with a 3.0 GHz CPU and 64 GB memory was 114 sec for the initial step of preparing the mouse network data, which only has to be done once, and then 409 sec (*n*_*t*_ = 2) or 824 sec (*n*_*t*_ = 16) for obtaining results for the response time *τ*_*r*_ corresponding to 50% density remaining at the driver. Times for a MacOS 12.7.4 system with an Apple M1 Pro CPU and 32 GB memory were faster, 59 sec for the initial step and 322 sec for *n*_*t*_ = 2. The NetPert software is available under the BSD 2-Clause Simplified License at https://github.com/joelbaderlab/netpert_v1 (Release v1.0). The repository is also published on Zenodo with DOI 10.5281/zenodo.11177051 at https://doi.org/10.5281/zenodo.11177051 and at https://zenodo.org/doi/10.5281/zenodo.11177051. The repository includes a setup script to create a Conda environment and download databases of protein-protein and gene-regulatory interactions, a driver script, and sample inputs and outputs. In addition to NetPert, the repository also includes implementations of betweenness centrality and TieDIE.

Our implementation considers a single driver gene. Calculations with multiple drivers could be accomplished by creating a new source vertex with a directed edge to each desired driver. Although in principle this introduces a delay time for the density to flow from the single source to the driver genes, in practice large rate constants may be assigned to the source-driver edges so that the delay time is negligible compared to other network timescales.

## Results

### Comparison with differentially expressed genes

The NetPert method and two alternative methods, betweenness centrality (BC) and TieDIE [23], were applied to the *Twist1*-driver mouse model of epithelial cell dissemination in breast cancer with 182 significantly differentially expressed genes. When all mouse interactions in the IID and TRRUST databases were included, the network consisted of the driver *Twist1*, 166 of the 182 differentially expressed genes and 16,389 intermediate genes (Table 1). Of the 182 differentially expressed genes, 32 had protein products that were known targets of small molecule compounds as reported by the Drug Repurposing Hub [7]. The driver TWIST1 was not targetable. The full network has 2,062 targetable intermediate proteins, a 63-fold increase in targetable proteins compared to proteins of differentially expressed genes. Although the full network contains 16,572 genes, only the 16,556 genes with at least one interaction are included in results tables and subsequent analysis (S5 Table). The remaining 16 genes are response genes without reported interactions.

**Table 1:** Proteins targetable by drugs in the Drug Repurposing Hub.

Category	Proteins		Drugs
	Total	Targetable	
Driver and response	183	32	259
Intermediates	16,389	2062	4259
Entire network	16,572	2094	4327

**Driver and Response:** Driver *Twist1* and differentially expressed response genes. Although counts include 16 differentially expressed response genes without interactions, these genes were not included in the analysis. **Intermediates:** Genes with at least one protein-protein or gene-regulatory interaction, excluding driver and response genes. **Entire network:** Driver, response, and intermediate proteins. The number of driver and response proteins plus the number of intermediate proteins sum to the number of proteins in the entire network. Of the 4327 total drugs considered, 191 have targets in both the driver/response and intermediate protein subsets.

By increasing the number of potential targets, NetPert also increases the number of small molecules or drugs that can be considered, essentially including all possible druggable targets. Thus, while the differentially expressed genes are accessible by 259 small molecules, the rest of the network increases the number of drug candidates to 4,327 small molecules (Table 1). Therefore, NetPert greatly expands the number of candidate targets, compared to those characterized by differential expression, that may be prioritized for novel small molecule development or validated by existing small molecules or genetic perturbations, including shRNA or CRISPR assays.

Certainly, most of these small molecules will not be relevant to the biology in question; the goal of NetPert is to prioritize the subset that are relevant. Biological networks have small world properties [48–50]: genes and proteins separated by only a small number of interactions from a driver or response gene might be peripheral or unrelated to the biological response. The value of NetPert is to prioritize the candidate targets across the entire network. As a benchmark, we also calculated priorities using a standard approach of ranking differentially expressed genes by log fold-change. This ranking by log fold-change implicitly incorporates a therapeutic hypothesis: inhibiting up-regulated genes is more likely to have an effect than inhibiting down-regulated genes. We did not attempt to incorporate a similar therapeutic hypothesis into NetPert because the activating or repressing activity of biological interactions can be context-dependent, and many remain unknown.

**Fig 2:**
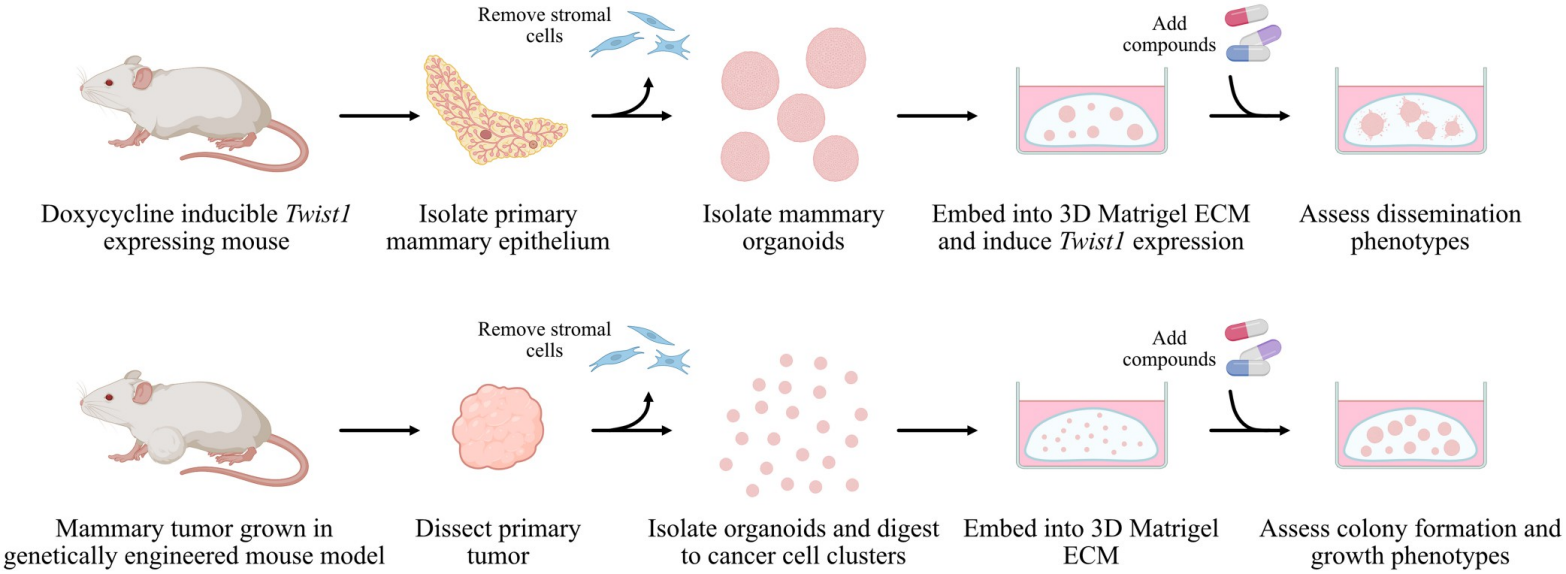
Wet-lab assays with genetically engineered mouse models of breast cancer were used to test the ability of chemical perturbations to stop metastatic phenotypes. Top panel, dissemination assays: Mammary tissue from a *Twist1*-inducible mouse model was dissected and stromal cells were removed to generate epithelial organoids. Larger organoids of 200–500 cells were isolated and embedded into 3D Matrigel growth medium. Following activation of *Twist1*, organoid dissemination upon treatment with compounds was quantified relative to untreated controls [21]. Bottom panel, colony formation assays: Mammary tumors from a genetically engineered mouse model were dissected, depleted of stromal cells, and used to generate epithelial organoids. Organoids were digested to clusters of 2–10 cancer cells and embedded into 3D Matrigel. The ability of organoids to form colonies subsequent to treatment with compounds was quantified relative to untreated controls.

**Fig 3:**
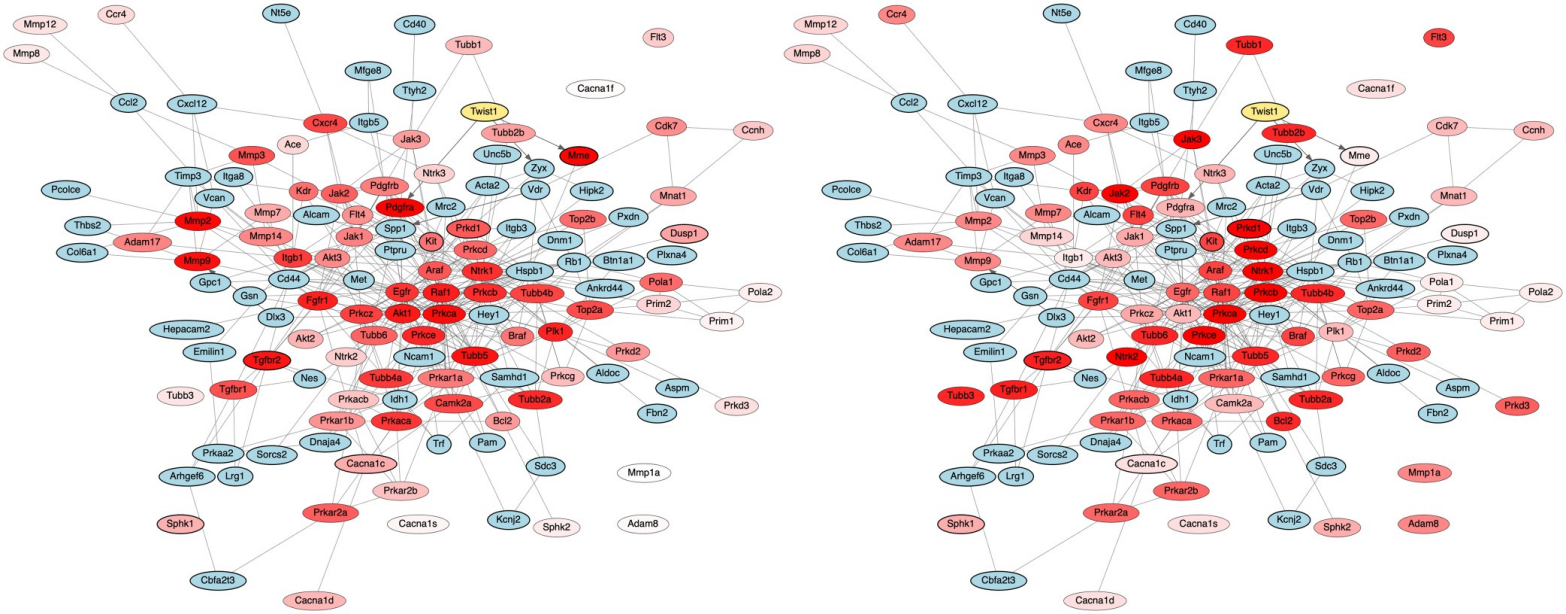
NetPert prioritization vs dissemination assay results. Subnetwork of intermediate proteins (shade of red) tested in the dissemination assay [21], driver *Twist1* (yellow, bold outline), differentially expressed response genes (shade of red, bold outline) that are either part of the network and tested in the assay, and differentially expressed response genes (blue, bold outline) that have a direct interaction with a tested intermediate protein. Gene-regulatory interactions (solid line with arrow head). Protein-protein interactions (solid line). Left panel: NetPert rankings of tested intermediates and differentially expressed response genes that are part of the network (shade of red). The deeper the red shade, the higher the ranking. Right panel: Dissemination assay results of tested intermediates and differentially expressed response genes that are part of the network (shade of red). The deeper the red shade, the greater the inhibition of dissemination by small molecules targeting a protein.

Rankings from NetPert were then compared with independent wet-lab assay results testing the ability of chemical perturbations to stop dissemination [21]. The wet-lab assays used the *Twist1*-driver mouse model of epithelial cell dissemination to generate organoids and measured the ability to disseminate in the medium (Fig 2). Networks with genes colored by NetPert ranking and by experimental results are qualitatively similar (Fig 3). For a quantitative assessment, we calculated Spearman rank correlations of predicted ranks versus assay results and then assessed statistical significance using two-sided tests. Three groups of proteins were considered individually: the driver and response genes; the intermediates; and the entire network of driver, intermediate, and response genes (Table 2). Rankings provided by NetPert across the entire network were correlated with wet-lab results with very high statistical significance (rank correlation 0.33, *p* = 0.0032, Table 2), and rankings for only the intermediates were also significant (rank correlation 0.34, *p* = 0.0036, Table 2). While these are single-test p-values, they remain significant at the conventional 0.05 family-wise error rate after accounting for 8 total approaches used (two NetPert methods versus three groups of targets, one BC test, and one TieDIE test).

**Table 2:** Prioritization results, NetPert versus differential expression.

Category	Driver and Response		Intermediates		Entire network	
Total proteins	183		16,389		16,556	
Prioritization	log-fc	NetPert	log-fc	NetPert	log-fc	NetPert
Tested proteins	7	7	64	72	71	79
Tested drugs	13	13	21	21	24	24
Correlation	−0.50	0.14	0.03	**0.34**	−0.05	**0.33**
P-value	0.25	0.76	0.81	**0.0036**	0.68	**0.0032**

**Driver and Response:** Driver *Twist1* and differentially expressed response genes. **Intermediates:** Genes with at least one protein-protein or gene-regulatory interaction, excluding driver and response genes. **Entire network:** Driver, response, and intermediate genes. Differentially expressed response genes without an interaction were not included in the analysis. **Tested proteins:** Proteins tested in the pharmacological inhibition assay of dissemination [21]. **Tested drugs:** Small molecules tested in the pharmacological inhibition assay of dissemination. **Prioritization:** Method used to rank protein targets, either log-fc (direct ranking by log fold-change of differential expression) or NetPert. **Correlation:** Spearman rank correlation of prioritization with dissemination assay results. **P-value:** Two-sided, single-test p-value for rank correlation. Text in **bold font** indicates statistical significance at 0.05 family-wise error rate after accounting for 8 total tests (two NetPert methods and three groups of targets, one BC test, and one TieDIE test).

In contrast, direct ranking by log fold-change for the entire network did not have significant correlation with the assay results, and the nominal direction was negative (rank correlation −0.05, *p* = 0.68, Table 2). Direct ranking of the intermediates alone was also not significant (rank correlation 0.03, *p* = 0.81, Table 2). While most analyses use rankings rather than direct scale for easier comparisons across methods, we note that Pearson correlations between log fold-change and dissemination assay results are negative and not significant, whereas Pearson correlations between log-scale NetPert weights and dissemination assay results are significant (S1 Fig). These results demonstrate that NetPert is effective in using network information to identify candidate drug targets based on their ability to perturb connectivity between driver and response genes.

We also investigated performance of rankings of the driver and response genes alone. Surprisingly, rankings calculated from differential expression were negatively correlated with assay results, although not statistically significant (rank correlation −0.50, *p* = 0.25, Table 2). Rankings generated by NetPert were modestly correlated with wet-lab results, but lacked statistical significance (rank correlation 0.14, *p* = 0.76, Table 2).

Two hypotheses may explain why driver and response rankings are less correlated with experimental results than rankings of the entire network. First, regarding the log fold-change results, it may be that significant differential expression strongly implicates a gene in a pathway, and once this threshold is passed the quantitative fold-change is less informative. Furthermore, the genes contributing to the log fold-change results were selected using a stringent p-value threshold [1, 21], which restricted attention to a small number of possible targets. The experimental studies did not assess robustness to less stringent thresholds.

Second, response genes may represent individual effectors. If these are pathway end-points, then inhibiting one is unlikely to inhibit many others. In contrast, intervening at an intermediate point closer to the driver may be more effective in inhibiting multiple end-points. This hypothesis is in concordance with our approach to exclude the weight of a driver or response gene on its own path. When we do include these self-terms (NetPert-Endpoints, Eq 2), rankings for the entire network are less correlated with experimental results (rank correlation 0.23, *p* = 0.039).

One complication of this analysis is that many drugs are reported to target multiple proteins, and similarly many proteins are targeted by multiple drugs. In the tested set of 83 proteins and 24 drugs, each protein was targeted by an average of 1.4 drugs, and each drug was reported to target an average of 4.9 targets. Identifying which subset of putative targets are the effective targets for an assay in principle could be accomplished using genetic rather than chemical perturbations, as has been done for some of the genes in this system as additional biological validation [21]. Of the 13 drugs targeting driver and response genes and 21 drugs targeting intermediates, a subset of 10 drugs were shared.

### Robustness to the response time and convolution time-step

**Fig 4:**
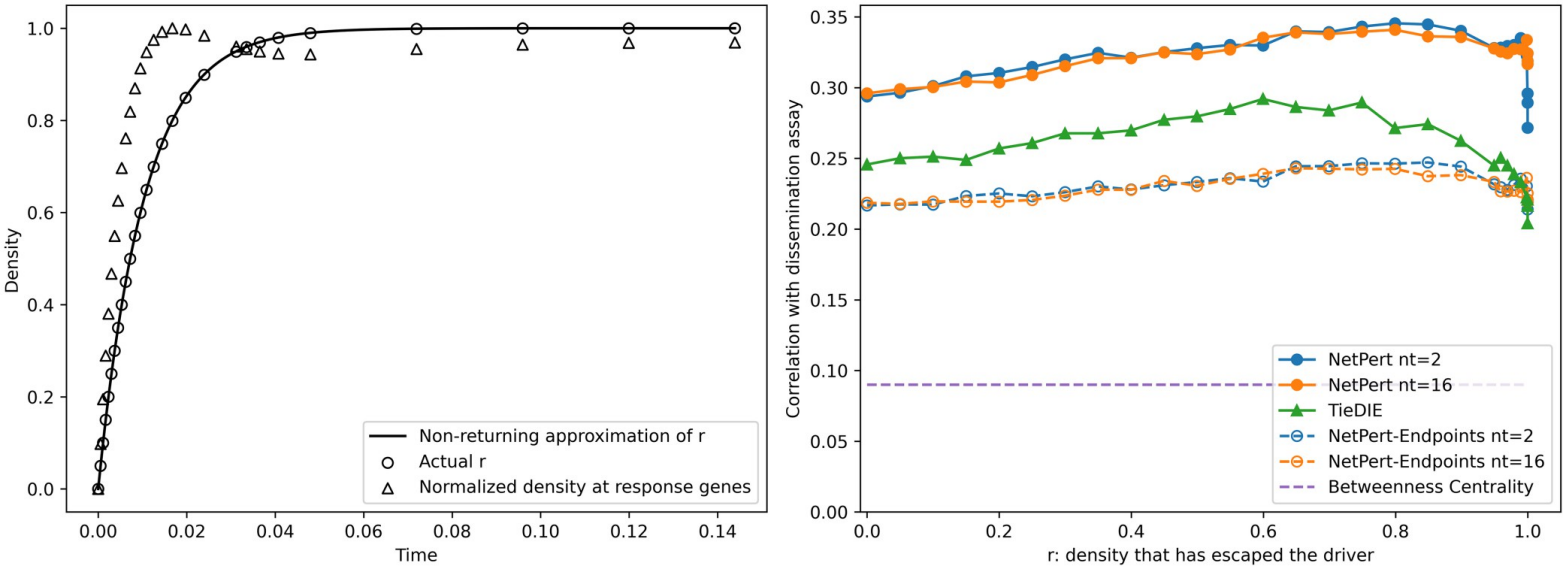
Diffusion time. Left panel: The density that has left the driver (open circles) and its approximation by the non-returning approximation (solid line) are shown as a function of the diffusion time. The total density at response genes (open triangles) is shown normalized to its maximum value, which occurs when approximately 80% of the density has left the driver. Right panel: Correlation with dissemination assay results [21] versus amount of density that has left the driver for NetPert, NetPert-Endpoints, TieDIE, and betweenness centrality.

The only free parameter in our computational model is the time at which the response is estimated, which can also be interpreted as the amount of density that has left the driver. We evaluated the non-returning approximation for the amount of density that has left the driver by comparing the actual density at time −(1/*d*_*i*_) ln(1 − *r*) with the approximate value *r* (Fig 4). The approximation is accurate from short times, *r* = 0, through long times, *r* = 0.9998, when most density has left the driver. Note that with 16,556 genes in the entire network, the ergodic long-time expectation of equal density at each vertex is *r* = 16,555/16,556, or 0.99994. The density at the response genes (excluding the driver) is initially 0, then rises to a maximum as density leaves the driver and washes over the response genes, and then falls again as the density randomizes throughout the network (Fig 4). The density is maximum at the response genes when approximately 80% of the density has left the driver.

We investigated method robustness by calculating Spearman rank correlation of rankings with dissemination inhibition assay results over a range of diffusion times (Fig 4). The correlation coefficients for NetPert and NetPert-Endpoints were robust to diffusion time and the number of time steps, changing by less than 28% from *r* = 10^−10^ (the choice *r* = 0 formally gives zero values for all genes) through *r* = 0.9998 (Fig 4, data provided in S4 Table). To avoid over-fitting, we selected *r* = 0.5 as a reasonable choice rather than attempting to optimize *τ*_*r*_. We note, however, that the best performance occurs for *r* ≈ 0.8, when 80% of the density has left the driver, and also the time of maximum density at the response genes. These results suggest that an improved criterion for selecting *τ*_*r*_ may be to select the time when the density at response genes is maximum.

We also investigated the convergence of NetPert with the number of time intervals used to calculate the convolutions, Eqs 2 and 3. For a given diffusion time *τ*_*r*_, we selected *n*_*t*_ equally spaced intervals of *τ*_*r*_/*n*_*t*_. Rankings generated with *n*_*t*_ = 2 and *n*_*t*_ = 16 are nearly identical in performance for correlation with wet-lab assays (Fig 4). Results from NetPert methods were generated with *n*_*t*_ = 2 unless described otherwise.

### Comparison with betweenness centrality

The NetPert method was compared with betweenness centrality (BC) [9, 10] as a standard benchmark for identifying network connectors. BC scores and rankings were generated for the *Twist1*-driver mouse model of epithelial cell dissemination in breast cancer. Genes that did not lie on any shortest paths from driver to response genes were assigned tied ranks at the end of the list. Rankings were then compared with independent wet-lab assay results testing the ability of chemical perturbations to stop dissemination in the same biological system [21]. Rankings provided by BC were modestly correlated with wet-lab results, but lacked statistical significance (rank correlation 0.09, p = 0.41, Spearman rank correlation two-sided test, Table 3).

**Table 3:** Prioritization results, NetPert versus betweenness centrality (BC) and graph diffusion (TieDIE).

Prioritization	NetPert	BC	TieDIE
Tested proteins	79	79	79
Tested drugs	24	24	24
Correlation	**0.33**	0.09	0.28
P-value	**0.0032**	0.41	0.013

**Tested proteins:** Proteins tested in the pharmacological inhibition assay of dissemination [21]. **Tested drugs:** Small molecules tested in the pharmacological inhibition assay of dissemination. **Prioritization:** Method used to rank protein targets in the entire network, including driver, response, and intermediate genes. Differentially expressed response genes without an interaction were not included in the network. **Correlation:** Spearman rank correlation of prioritization with dissemination assay results. **P-value:** Two-sided, single-test p-value for rank correlation. Text in **bold font** indicates statistical significance at 0.05 family-wise error rate after accounting for 8 total tests (two NetPert methods and three groups of targets, one BC test, and one TieDIE test).

As the most obvious difference between NetPert and BC is the ability to rank genes that are not on shortest paths, we separately analyzed the tested genes that were on at least one shortest path and those not on any shortest paths. Of the 16,556 total genes in the network, 1066 are on a shortest path, and 22 of these were tested. For the remaining 15,490 non-shortest-path genes, 57 were tested. The shortest-path targets had dissemination scores of 41 ± 32, somewhat better than the dissemination scores of 50 ± 33 for non-shortest path targets, but not significantly different (*p* = 0.28, two-sided unequal-variance *t*-test).

For the genes on shortest paths, the NetPert rank correlation with assay results was −0.15 (*p* = 0.50, two-sided test), and the BC correlation was −0.18 (*p* = 0.41). For genes not on shortest paths, the NetPert correlation was 0.45 (*p* = 0.0004), and the BC correlation was necessarily 0 because these genes were all tied. Our interpretation is that NetPert outperforms BC because it can rank genes that are not on shortest paths, and non-shortest-path targets are a sizable fraction of targets overall.

The lack of statistical significance and negative correlation for genes on shortest paths is surprising, although similar to the lack of statistical significance and negative correlation for genes ranked by log-fold change (Table 2). Our interpretation is that network-based methods are capable of identifying shortest-path genes as important, but have difficulty in relative rankings within this category, at least for this data set.

### Comparison with graph diffusion

The NetPert method was also compared with the Tied Diffusion through Interacting Events (TieDIE) method [23], which identifies genes that link forward diffusion from the driver to backward diffusion from the response genes. The min function is used to identify genes that are strongly linked to both. The TieDIE method was applied to the IID and TRRUST network to prioritize all genes for the *Twist1* driver mouse model of epithelial cell dissemination in breast cancer with 182 differentially expressed genes. Rankings were then compared with independent wet-lab assay results testing the ability of chemical perturbations to stop dissemination in the same biological system [21], yielding a Spearman rank correlation of 0.28 (*p* = 0.013, two-sided test, Table 3). While the TieDIE performance was better than BC and the p-value was significant for a single test, it was not significant at the conventional 0.05 family-wise error rate after accounting for 8 total tests (six NetPert tests, one BC test, and one TieDIE test) and not as good as NetPert (Table 3).

We then investigated how NetPert and TieDIE ranked nodes categorized by their connections to driver (‘D’) and response (‘R’) genes. Intermediates falling on at least one path of length 2 between the driver and a response gene were categorized as ‘DIR’ (72 genes), and intermediates on at least one path of length 3, but not paths of length 2, were categorized as ‘DIIR’ (3,264 genes) (see S5 Table for a full listing of all 16,556 genes). The NetPert method gave high ranks to the DIR genes, ranks 1 through 76. NetPert ranked two DIIR genes higher than some DIR genes: *G3bp2* ranked 73, and *Kmt5a* ranked 75. Two genes counted as ‘R’ are on paths of length 2 and would otherwise be grouped with DIR genes: *Zyx* ranked 7, and *Mme* ranked 56.

While NetPert ranked the 72 DIR genes highly (ranks 1 through 76), TieDIE ranked DIR genes less highly (rank 3 through rank 1,662). The DIR gene ranked lowest by TieDIE (*Rad23a* ranked 1,662) was ranked behind 1,533 DIIR genes, 29 R genes, the driver, and 27 I genes. For the 3,264 DIIR genes, NetPert ranks were 73 (99.6 percentile) through 6,300 (62 percentile), compared to TieDIE ranks of 13 (99.9 percentile) through rank 13,939 (16 percentile).

We also investigated the robustness of the TieDIE rankings to diffusion time. The correlation coefficients for the TieDIE method had a broad region of good performance, similar to NetPert, but were also consistently smaller than those for NetPert from *r* = 10^−10^ through *r* = 0.9998 (Fig 4). Similar to NetPert, we avoided over-fitting the TieDIE model by selecting *r* = 0.5 for all reported results.

In summary, for this dataset, NetPert performed better than a previous graph diffusion method, TieDIE, for ranking drug targets correlated with wet-lab assay results. The major difference between the NetPert and TieDIE methods is that NetPert uses a product form that follows from perturbation theory, whereas TieDIE uses a min function to link forward diffusion from the driver to backward diffusion from the response genes. A second difference is that NetPert uses a full convolution of perturbation times compared with a single calculation at the halfway time for TieDIE. Equivalent results for NetPert with 2 time intervals (equivalent to the single calculation of TieDIE) and 16 time intervals suggest that this difference is not as important (Fig 4). Overall, these comparisons suggest that response functions and perturbation theory motivated by physics-based systems can help guide the development of computational methods for analyzing biological systems.

### Performance across PPI networks

Performance variation across PPI networks was investigated by using the Search Tool for Recurring Instances of Neighbouring Genes (STRING) and the Human Reference Interactome (HuRI) as alternatives to IID as sources for PPIs; regulatory interactions were retained from TRRUST. STRING contains PPI data from experimental studies, protein complex and pathway knowledgebases, parsed full-text journal articles, and parsed summary texts from online catalogs [25]. HuRI is a dataset consisting of PPIs detected in high-throughput yeast two-hybrid screens and extracted from the literature [26]. The STRING mouse network had 352,463 PPIs (Table 4) and the HuRI network, after mapping from human to mouse, had 85,302 PPIs. Both PPI networks are substantially smaller than the IID mouse PPI network, which had 539,606 PPIs.

**Table 4:** Network interaction counts.

	Mouse	Mouse and human
IID	539,606	1,420,873
STRING	352,463	932,886
HuRI	0	85,302
TRRUST	6,490	15,260

**Mouse:** The number of mouse-only interactions from the specified database. **Mouse and human:** The union of mouse-only and human-mapped-to-mouse interactions from the specified database. **IID:** PPIs from the Integrated Interactions Database [24]. **STRING:** PPIs from the Search Tool for Recurring Instances of Neighbouring Genes [25]. **HuRI:** PPIs from the Human Reference Interactome [26]. **TRRUST:** gene-regulatory interactions from Transcriptional Regulatory Relationships Unraveled by Sentence-based Text mining [27].

The NetPert, BC, and TieDIE methods were applied to each network to rank all genes for the *Twist1*-driver mouse model of epithelial cell dissemination in breast cancer. Rankings were then compared with independent wet-lab assay results testing the ability of chemical perturbations to stop dissemination in the same biological system [21]. Spearman rank correlations of predicted ranks versus assay results were calculated. The performance of each method on the smaller STRING and HuRI mouse networks decreased from the IID mouse network (Table 5). NetPert outperformed both TieDIE and BC on both smaller networks, as it did on the larger IID network.

**Table 5:** Prioritization results across PPI networks.

	NetPert		TieDIE		BC	
	M	MH	M	MH	M	MH
IID + TRRUST	0.33	0.33	0.28	0.31	0.09	0.01
STRING + TRRUST	0.09	0.22	0.05	0.18	0.03	0.01
HuRI + TRRUST	0.19	0.14	0.07	0.11	0.08	-0.05

**M:** Spearman rank correlation of the specified prioritization with the dissemination assay results [21] using the mouse-only interactions from the specified databases. **MH:** Spearman rank correlation of the specified prioritization with the dissemination assay results using the union of the mouse-only and human-mapped-to-mouse interactions from the specified databases. **IID + TRRUST:** Network of PPIs from the Integrated Interactions Database [24] and gene-regulatory interactions from Transcriptional Regulatory Relationships Unraveled by Sentence-based Text mining [27]. **STRING + TRRUST:** Network of PPIs from the Search Tool for Recurring Instances of Neighbouring Genes [25] and gene-regulatory interactions from TRRUST. **HuRI + TRRUST:** Network of PPIs from the Human Reference Interactome [26] and gene-regulatory interactions from TRRUST.

Since performance was best for the largest network, we investigated robustness to even larger networks obtained by our own automated mapping of human PPIs to mouse orthologs, adding these to the mouse interactions directly provided by IID, STRING, and TRRUST, using the same human-to-mouse conversion process we used for HuRI. Each interaction network increased in size substantially (Table 4). Both NetPert and TieDIE generally performed better with the larger mouse plus human networks rather than the mouse-only networks. NetPert performed somewhat better than TieDIE for each network and performed better when TRRUST interactions were limited to mouse-only (Table 5). In contrast, BC performed best for the mouse-only network, and poorly overall.

Coverage of protein interactions by experimental methods remains limited [51–53]. Augmenting mouse-only interactions with human-to-mouse interologs [54] could recover true interactions missing from mouse-only data, but could also introduce false-positive interactions that occur in human but not in mouse. Our results indicate that NetPert and TieDIE are robust to errors in network data, with results generally improving for larger networks that may include more false-positive edges. In contrast, BC is less robust to possible errors in network data.

### Testing non-differentially-expressed intermediates in a metastatic outgrowth mouse model

An intended use of the NetPert method is to prioritize intermediate genes that connect the driver gene to the differentially expressed response genes. As above, ‘D’ refers to the driver *Twist1*, ‘R’ refers to response genes, and ‘I’ refers to intermediates, all genes in the network except for the D and R genes. Intermediates having direct connections to the driver and at least one response gene were categorized as ‘DIR’ (72 genes); intermediates connected directly to the driver but not to a response gene were ‘DI’ (22 genes); intermediates connected directly to a response gene but not the driver were ‘IR’ (4598 genes); and intermediates connected to neither the driver nor a response gene were ‘I’ (11,697 genes). A full listing of rankings, categories, and assay results is available for all 16,556 genes (S5 Table).

Both driver and intermediate genes can control the activity of multiple downstream signaling and gene-regulatory pathways. Accordingly, the genes we identified as required for early stages of metastasis, invasion and dissemination, might also be required for later stages of metastasis, including outgrowth in distant organs. We therefore evaluated whether the inhibition of the activity of the gene products prioritized by NetPert could also disrupt metastatic outgrowth. Since 72 of the 76 highest-ranked genes were DIR genes, these genes are the focus of validations reported here. The median rank of the DIR genes was 37.5, substantially better than the median rank of differentially expressed genes (R genes), which was 7041.5. The dissemination assay prioritized differentially expressed genes and consequently only tested a single DIR target, PDGFRA [21]. The targeting compound was GNF-5837, a tropomyosin receptor kinase inhibitor discovered by the Genomics Institute of the Novartis Research Foundation [55]. This molecule was moderately active, reducing dissemination to 73% of untreated control, but was not among the most active in the assay.

Of the 72 DIR genes, the Drug Repurposing Hub lists compounds for 22 of the corresponding proteins. Compounds available from our in-house libraries targeted a subset of 16 proteins. These were tested using a colony formation assay relevant to the outgrowth of micrometastatic lesions into macroscopic tumors. Small clusters of cells were embedded into a 3D extracellular matrix that is similar in composition to the environment of metastatic sites. The ability of a cluster to grow *ex vivo* phenocopies metastatic success *in vivo* [3, 56, 57]. The colony formation assay was selected because it was more amenable to scale-up and because it permitted testing of the ability to block an additional step in the process of metastasis.

Of the 16 proteins tested, we found 7 to be highly effective targets, reducing colony formation to 1% or less of untreated control: BRD4, RELA, CDK9, and four histone deacetylases (HDACs), HDAC1, HDAC2, HDAC3, and HDAC6. Targeting two other proteins, STAT3 and TUBG1, reduced colony formation to 10–15% of untreated control. Most of the other targets were at least moderately effective, with colony formation at 30–70% of untreated control.

**Fig 5:**
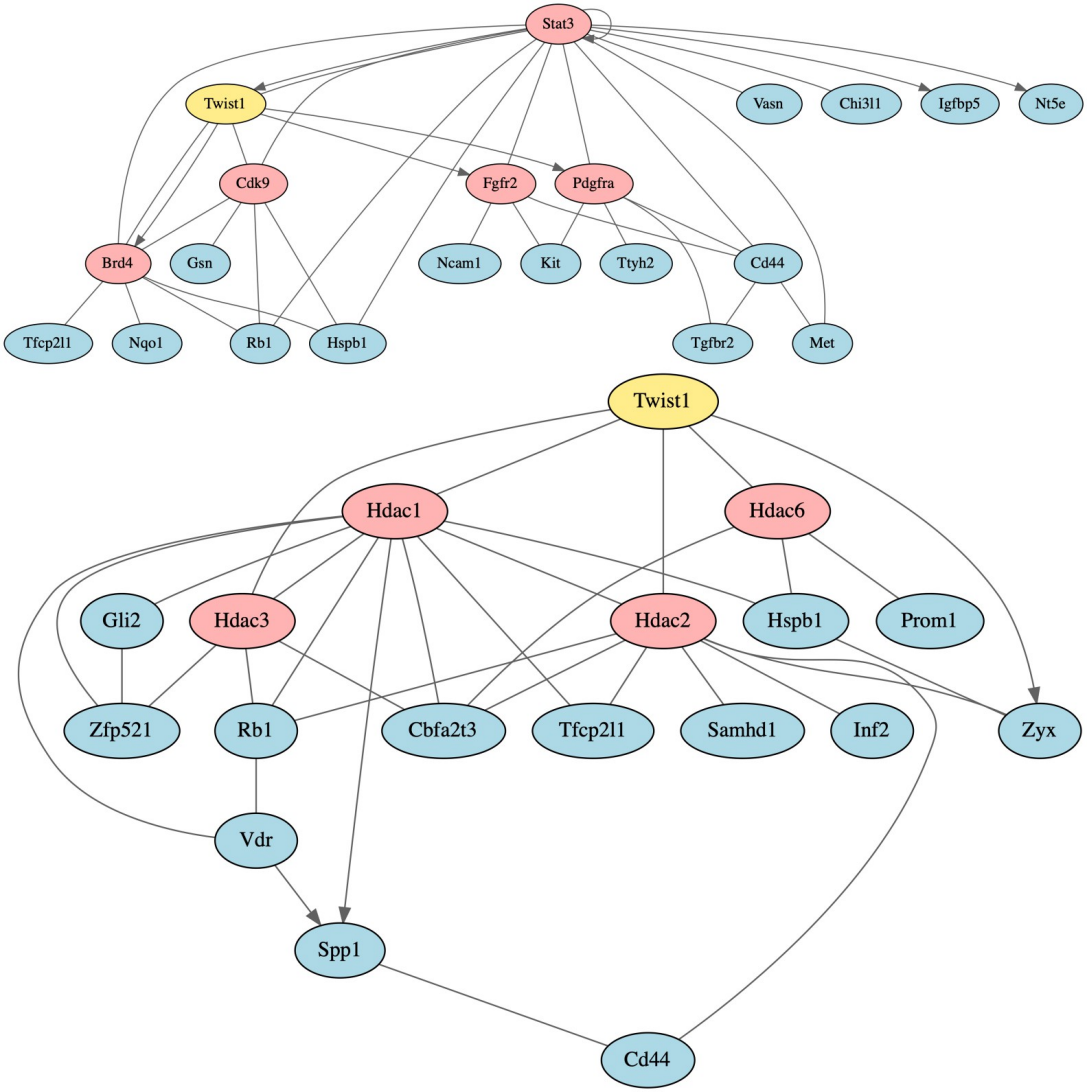
JAK/STAT signaling and HDAC subnetworks. The protein TWIST1 (yellow), a driver of metastatic phenotypes, signals through non-differentially-expressed intermediates (red) to cause differential expression of response genes (blue). Lines indicate protein-protein interactions and directed arrows represent gene-regulatory interactions. Top panel: In a colony formation assay, compounds targeting BRD4 and CDK9 eliminated colony formation entirely. Compounds targeting FGFR2 reduced colony formation to 34–74% of untreated control; compounds targeting PDGFRA reduced colony formation to 34–67% of untreated; and a compound targeting STAT3 reduced colony formation to 10% of untreated. Bottom panel: Compounds targeting HDAC1, HDAC2, HDAC3, and HDAC6 eliminated colony formation entirely.

Several of the effective targets form an intermediate layer in a JAK/STAT signaling subnetwork (Fig 5). This network contains STAT3, a master regulator of many processes, ranked 11 by NetPert. Unfortunately, clinical trials of human STAT3 inhibitors have revealed positive and negative feedback loops with RAS/RAF signaling that have hindered development [58, 59]. The STAT3 inhibitor tested was WP1066, a compound developed for hematologic malignancies [60], currently in a Phase I clinical trial for malignant brain tumors in children [61]. This inhibitor reduced colony formation to 10% of untreated control.

Underneath STAT3 are two separate pathway branches, one involving signaling intermediates BRD4 (ranked 74 by NetPert) and CDK9 (ranked 55), and the other involving intermediates FGFR2 (ranked 6) and PDGFRA (ranked 3) (Fig 5). The BRD4 protein (bromodomain-containing protein 4) regulates chromatin structure, and interactions between TWIST1 and BRD4 contribute to tumorigenesis in breast cancer [62]. In human breast cancer cell lines, knockdown or small-molecule inhibition of *BRD4* reduces migration and invasion phenotypes [63]. The CDK9 protein (cyclin-dependent kinase 9) functions in transcriptional regulation. Colony formation was eliminated entirely by two inhibitors of CDK9 and three inhibitors of BRD4, including the FDA-approved drug fedratinib that inhibits BRD4 and JAK2 [64]. Three additional inhibitors of BRD4 reduced colony formation to 1–5%.

In contrast, inhibiting the other pathway branch was not as effective. FDA-approved drug regorafenib [65], targeting FGFR2 and PDGFRA, reduced colony formation to 74%. The FDA-approved drug sunitinib, targeting PDGFRA, reduced colony formation to 67%. Finally, FDA-approved drug ponatinib [66], a pan-FGFR inhibitor with activity against human FGFR2 and PDGFRA, reduced colony formation to 34%.

The HDAC proteins were also highly-ranked effective targets, interacting with TWIST1 and many response genes (Fig 5). The histone deacetylases HDAC1 (ranked 37), HDAC2 (ranked 39), HDAC3 (ranked 24), and HDAC6 (ranked 32) are targets of the inhibitors trichostatin A, dacinostat, belinostat, and panobinostat. These HDAC inhibitors eliminated colony formation, and other HDAC inhibitors reduced colony formation to 1–5% of control. In human breast cancer, *HDAC1*, *HDAC2*, and *HDAC3* have been found to be differentially expressed [67]. Furthermore, *HDAC2* and *HDAC3* are strongly expressed in tumor subgroups with more aggressive features, such as less differentiated tumors and negative hormone receptor status [67].

### Highly-ranked intermediates

**Fig 6:**
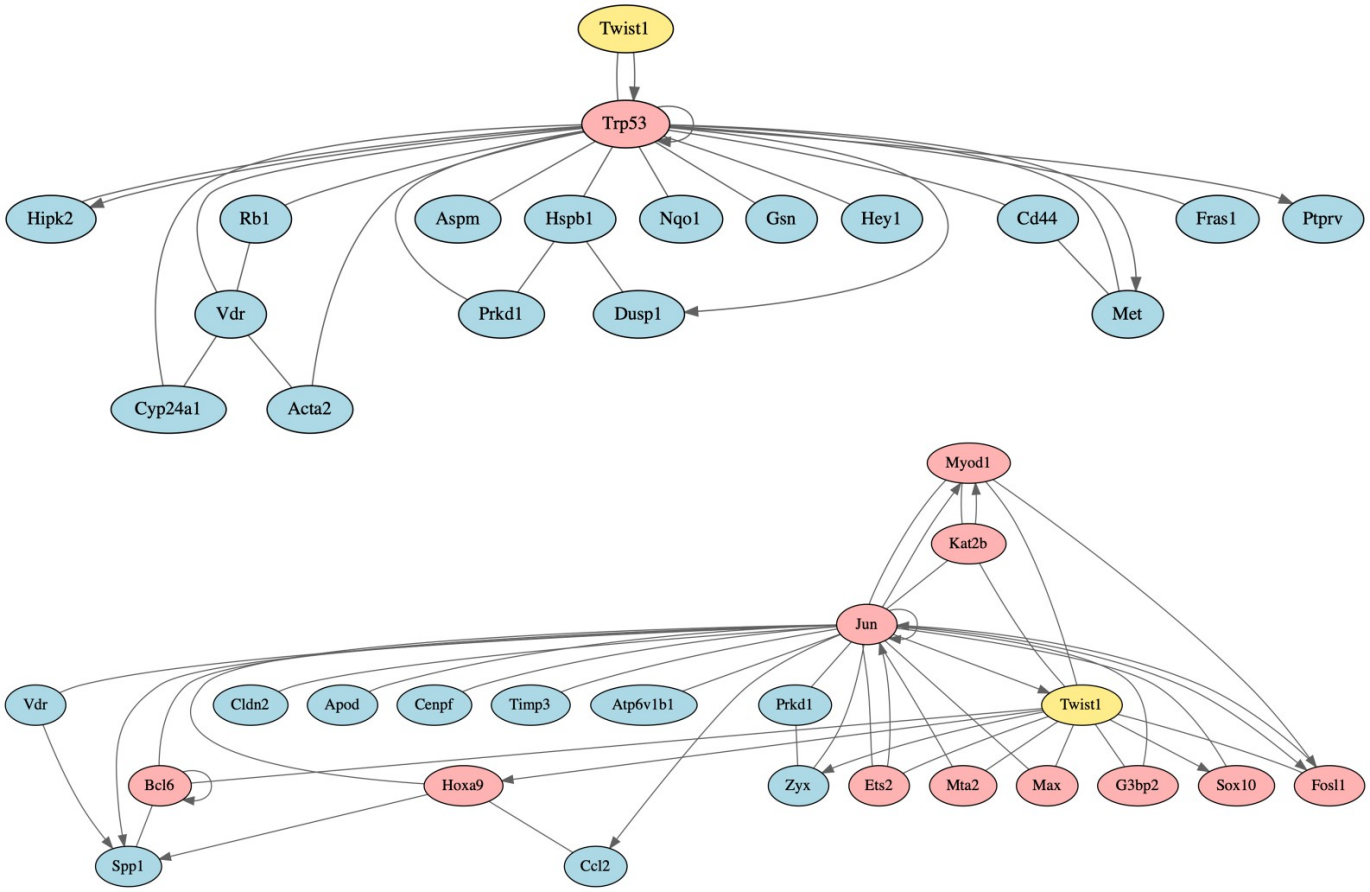
Subnetworks of TRP53 and JUN. Driver *Twist1* (yellow). Gene-regulatory interactions (solid line with arrow head). Protein-protein interactions (solid line). Top panel: *Trp53* (red); the differentially expressed genes (blue) that are TRP53 targets or proteins interact with TRP53. Bottom panel: *Jun* (red); the top 10 differentially expressed genes (blue) ranked by NetPert that interact with *Jun* and are sensitive to *Jun* perturbations with *Twist1* as the driver; and the top 10 intermediate genes (red) ranked by NetPert that interact with *Twist1* and *Jun* that *Jun* is sensitive to with *Twist1* as the driver and *Jun* as the response.

Ranked 48 overall by NetPert was *Trp53*, the ortholog of the human tumor suppressor gene *TP53*. The *Trp53* gene is a regulatory target of TWIST1, and the protein interacts with TWIST1 and 16 differentially expressed genes and gene products, making *Trp53* a DIR gene (Fig 6). In human breast cancer, *TP53* mutations are frequent and associated with more aggressive disease and worse overall survival [68, 69]. In mice, *Trp53* mutations cause tumors that resemble human breast cancers, particularly triple negative breast cancer (TNBC) [70]. At least 9 compounds in the Drug Repurposing Hub target TP53. The mouse protein TRP53 was not tested in either assay, however.

The proto-oncogene *Jun* (ranked 120) has no direct interaction from TWIST1 but is a transcriptional regulator of the *Twist1* gene (Fig 6). The transcription factor JUN interacts directly with 14 differentially expressed genes and proteins, making *Jun* an IR gene. One of the differentially expressed genes whose protein interacts with JUN is *Prkd1*, which was shown to be required for *Twist1*-induced dissemination [21]. If we consider TWIST1 the driver and JUN a single response gene, then two of the top ten intermediates ranked highest by NetPert are FOSL1 and ETS2. The FOSL1 protein is a member of the Fos gene family and dimerizes with members of the JUN family, resulting in the formation of Activator Protein-1 (AP-1), a transcription factor complex that binds DNA at AP-1 specific sites at the promoter, enhances regions of target genes, and converts extracellular signals into changes of gene expression [71, 72]. The transcription factor ETS2 has context-dependent oncogenic and tumor suppressor function, dependent in part on *TP53* mutation [73]. Expression of c-Jun, a component of AP-1, has been observed at the invasive front of breast tumors [74]. In mouse models, *Jun* contributes to ErbB2-induced mammary tumor cell invasion and self-renewal [75]. The protein JUN was not tested in the dissemination assay, but at least 4 compounds in the Drug Repurposing Hub target it.

**Fig 7:**
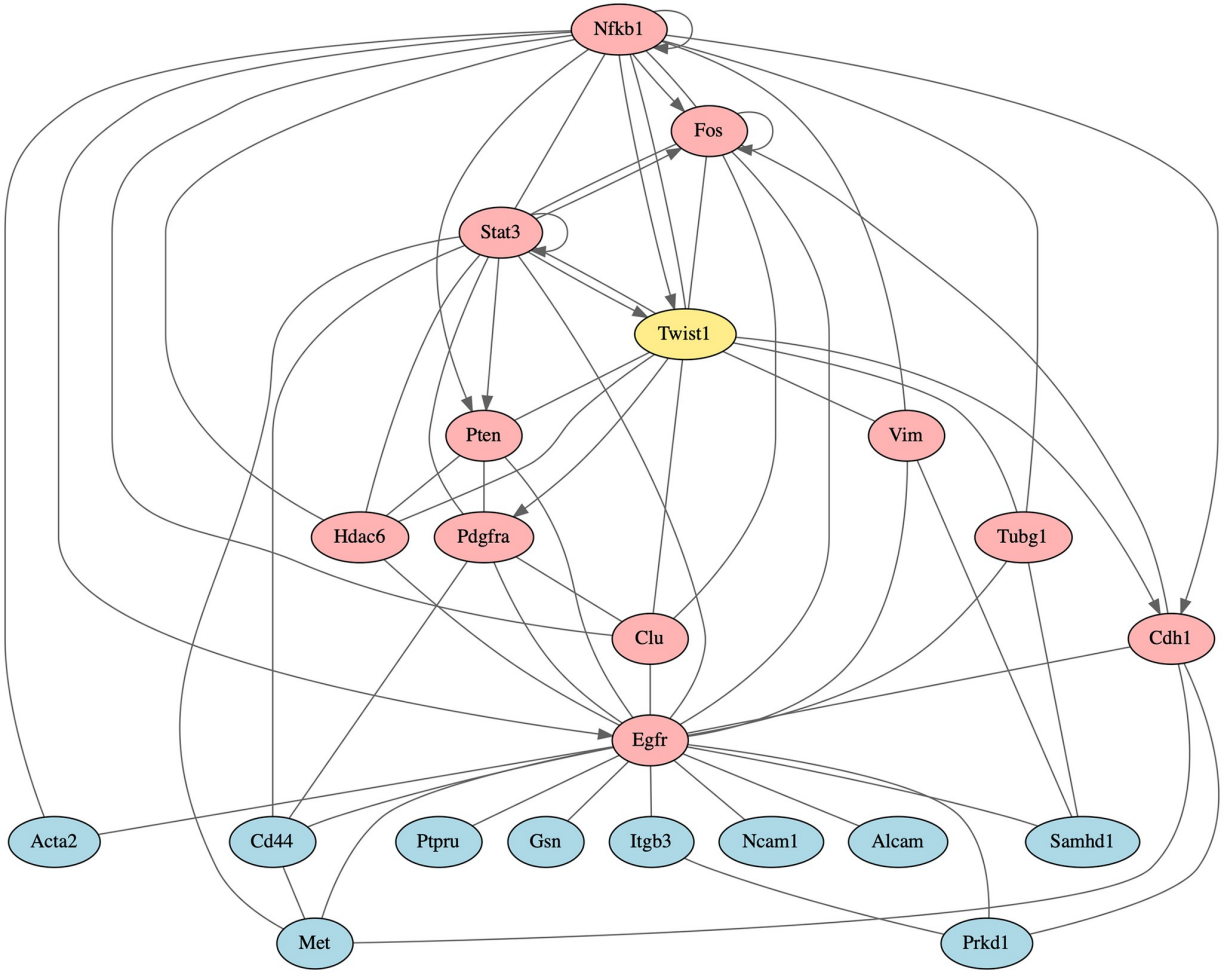
Subnetwork of EGFR. *Egfr* (red); driver *Twist1* (yellow); the top 10 differentially expressed genes (blue) ranked by NetPert that interact with *Egfr* and are sensitive to *Egfr* perturbations with *Twist1* as the driver; and the top 10 intermediate genes (red) ranked by NetPert that interact with *Twist1* and *Egfr* that *Egfr* is sensitive to with *Twist1* as the driver and *Egfr* as the response. Gene-regulatory interactions (solid line with arrow head). Protein-protein interactions (solid line).

The gene *Egfr* (ranked 354) interacts with response genes or proteins such as PRKD1, CD44, and MET, but not with TWIST1 directly, making *Egfr* an IR gene (Fig 7). The protein EGFR is a receptor tyrosine kinase with ligands from the epidermal growth factor family. Increased *Egfr* transcript levels and EGFR protein levels are associated with poor prognosis in various cancers [76]. Although activating mutations and gene amplifications of *Egfr* are low frequency occurrences in breast cancer, *Egfr* expression can be enhanced by increased gene copy number due to polysomy, and enhanced expression of *Egfr* in primary tumors is associated with increased metastasis and decreased survival of TNBC patients [77, 78].

The EGFR inhibitor genistein was a potent inhibitor of *Twist1*-induced dissemination, with an IC_50_ of 614 nmol/L. At a concentration of 1*μ*mol/L, genistein reduced dissemination to 33% of control [21]. The EGFR inhibitor erlotinib has been evaluated in two clinical trials, but was determined in both to not provide clinical benefit to breast cancer patients, even when incorporating knowledge of EGFR expression levels in the primary tumor [79, 80]. The protein EGFR is targetable by at least 53 small molecules in the Drug Repurposing Hub.

If we consider TWIST1 the driver and EGFR a single response gene, then STAT3, PDGFRA, HDAC6, FOS, CDH1, and VIM are in the top ten intermediates highest ranked by NetPert. We have already discussed STAT3, PDGFRA, and HDAC6. The FOS protein is another member of the Fos gene family, which we discussed with JUN. The CDH1 protein is E-cadherin, a cell adhesion effector gene whose loss of function increases dissemination while decreasing cell viability [3]. The VIM protein is vimentin, a type III intermediate filament expressed in mesenchymal cells. Vimentin expression correlates with tumor size and higher histological grade in breast cancers of young women [81]. Recent studies have shown that vimentin is required for invasion and metastasis in mouse models of TNBC [57].

## Discussion

Cancer is a disease of the genome, and molecular measurements hold promise for understanding cancer biology and guiding cancer therapy. Tumor DNA sequencing can reveal upstream drivers, and RNA sequencing can identify differentially expressed effectors. Exploiting this molecular information is crucial for the development of new therapeutics. Unfortunately, the intermediates between driver and effector genes are often missing or under-represented in differential gene expression analysis. This bias has skewed research towards terminal effectors that are differentially expressed, only a small number of which may be druggable. Furthermore, individual effectors may be pathway endpoints with limited influence on other signaling branches. In contrast, intermediates may regulate more pathway branches and be more likely to be druggable—if they can be identified despite their absence from differentially expressed gene lists.

The NetPert method uses perturbation theory to identify these intermediates by using biological interactions to interpret RNA sequencing data. Highly ranked intermediates often form a layer between the driver gene and multiple downstream signaling branches.

Candidates can be validated by small molecule inhibitors found in libraries such as the Drug Repurposing Hub [7] or direct genetic perturbations such as shRNA knockdown and CRISPR knockout. Validations using small molecules show that rankings provided by NetPert correlate much more strongly with experimental results than ranking by differential expression directly. Validations also support the hypothesis that intermediates that can interfere with pathways between driver and effectors may be better perturbation candidates than terminal effectors. In fact, we found that the fold-change of differentially expressed genes did not correlate with experimental performance. While this result is surprising, similar effects have been observed more generally. In a foundational yeast genomics study, for example, genes functionally identified as essential for growth in a defined environment lacked significant overlap with genes differentially expressed in that same environment [82]. The lack of concordance between gene essentiality and gene expression highlights the importance of augmenting RNA sequencing data with biological pathway information. A biological explanation for the lack of concordance is that differentially expressed effectors can provide overlapping function, creating a system robust to individual effector gene deletions—though potentially sensitive to deletions of intermediate regulators.

Several candidate targets for breast cancer metastasis identified by NetPert have been validated by experimental assays, are being evaluated in clinical trials, or are targets of existing compounds. The NetPert method identified several effective targets in the colony formation assay that are members of the JAK/STAT signaling subnetwork or HDAC protein family. The inhibition of STAT3 phosphorylation has reduced the expression of matrix metallopeptidases MMP2 and MMP9, which help enable breast cancer invasion and metastasis [83]. Disruption of Twist-BRD4 interaction by BET-specific inhibitors *in vitro* and *in vivo* have been shown to reduce WNT5A expression and inhibit tumorigenicity and invasion of basal-like breast cancer [62].

The HDAC proteins were validated by many HDAC inhibitors in the colony formation assay, and the genes *HDAC2* and *HDAC3* have been found to be differentially expressed in human breast cancer and strongly expressed in aggressive tumor subgroups [67].

The protein EGFR was highly ranked by NetPert and it was validated in the dissemination assay [21]. Enhanced expression of *Egfr* in primary breast tumors are associated with increased metastasis and decreased survival of TNBC patients [77, 78]. Interestingly, if we consider TWIST1 the driver and EGFR the single response gene, NetPert ranks STAT3, PDGFRA, and HDAC6 in the top ten intermediates that interact with both TWIST1 and EGFR. The NetPert method also highly ranked the proteins TRP53 and JUN, which were not tested in the experimental assays but are targets of known compounds. Mutations in *TP53* are frequent and associated with more aggressive forms of human breast cancer and worse overall survival [68, 69]. Expression of *JUN* has been observed in mitotic cells at the invasive front of breast tumors, indicating a potential role in both proliferation and invasion [74].

Colony formation validations used the MMTV-PyMT mouse model. Genetically engineered mouse models are an important resource for cancer research. Transcriptomic analysis classifies MMTV-PyMT as a luminal-type model, whereas other models are classified as basal-like [84]. Further exploration of the targets suggested by NetPert could involve establishing generality across different mouse models and patient-derived xenografts (PDXs), from organoids to whole animal studies, as we have done previously [3].

The main requirements of NetPert are experiments relating driver activity to differential gene expression, readily provided by RNA sequencing data, and catalogs of gene and protein interactions, available from public databases of measured and inferred protein-protein interactions and gene-regulatory interactions. In our applications, we treated network edges as having equivalent weights, yielding a semi-quantitative method with a single parameter representing the observation time after a pulse of activity from the driver. Prioritized rankings were robust to this single time. The semi-quantitative model could be refined by incorporating individual kinetic parameters for gene and protein interactions, direction-of-effect for activation versus repression, and therapeutic direction by considering that drugs typically down-regulate the activities of their targets. Kinetic parameters in the model could also be estimated from experimental measurements of the system under study, for example decay terms assessed by RNA velocity and protein velocity from single cell experiments, for potential systematic improvements [85, 86].

Different databases of protein interactions are available, with different strategies for including direct interactions versus protein complex co-membership and for mapping interactions across species. The NetPert method performed better than other methods in generating rankings that correlated with wet-lab assay results across all databases tested. While methods based on network dynamics were robust and generally improved for databases with more interactions, betweenness centrality was less robust and did not improve.

A possible limitation of our approach is that response functions are based directly on interaction databases, which are incomplete and noisy. Causal reasoning is an alternative route to developing computable network models [87]. Applications to biological networks have used causal reasoning and the do-calculus to create systems-level models [88–92]. Progress could also involve joint analysis of NetPert response functions with data from chemical or genetic perturbation assays. Comparisons could be used to improve confidence in mechanistic interactions supported by experimental data, prune interactions not supported by data, or clarify overall directionality of causal pathways.

The computational efficiency of NetPert is sufficient for genome-scale networks. The short-time limit of NetPert yields an expression similar to betweenness centrality (BC). Unlike BC, however, NetPert considers all intermediates, not just those on shortest paths from drivers to differentially expressed genes. Instead, NetPert considers all paths between driver and downstream response. A driver connected directly to a single response gene provides an example. Intermediates that connect from the driver to this response gene will be highly ranked by NetPert. The BC method, however, will be unable to rank these intermediates because the shortest path is the direct path. Overall, NetPert performed substantially better than BC.

The NetPert rankings are obtained by taking the derivative of a two-point response function to yield a three-point function as a convolution over two-point response functions, as guided by perturbation theory. We show that NetPert performs better than the graph diffusion method, TieDIE, which uses the min function to generate a three-point function from two-point functions [23].

Other approaches have been suggested for identifying network intermediates. The prize-collecting Steiner tree (PCST) problem, for example, is to find a set of connecting edges that optimizes a cost function, and exact solvers have been described [93]. Prize-collecting Steiner trees have been used productively to identify components of signaling pathways [94]. The PCST problem is somewhat different from our problem, however, because the PCST objective function effectively limits the number of intermediate vertices, whereas our goal is to provide a ranking for all vertices. Consider, for example, a network in which a single driver gene is connected to an intermediate gene, which in turn is connected to each of the response genes, and a second intermediate that is connected to all but one response gene. The NetPert approach would rank both intermediates highly. The PCST solution, however, would include the first intermediate and leave the second intermediate unranked. Thus, while the PCST approach has been successful for biological network analysis, it would require modifications for the problem considered here.

While the focus of this initial description of NetPert has been on single-vertex perturbations, drug combinations are often used in cancer therapy and drug repositioning [95–98]. Drug combinations can target multiple pathway arms, which are evident in NetPert results. A JAK/STAT subnetwork identified by NetPert, for example, includes a BRD4-CDK9 pathway arm and a FGFR2-PDGFRA pathway arm (Fig 5). A combination therapy targeting both arms could employ fedratinib and ponatinib, each already approved for use in leukemia. A natural extension of NetPert would be to use higher-order perturbation theory to predict synergistic multi-gene perturbations for combination drug therapies.

## Conclusion

The NetPert method uses perturbation theory to identify targets that can disrupt signaling from upstream driver genes to downstream effectors. Target rankings predicted by NetPert correlate stronger with the experimental effect of perturbing the targets than rankings directly from differential expression, suggesting intermediates that can interfere with pathways between driver and effectors are better perturbation candidates than terminal effectors. A short-time expansion of the NetPert perturbation theory shows a close connection to betweenness centrality. While betweenness centrality is limited to genes on shortest paths from the driver to the response, however, NetPert does not have this limitation and is more robust to noise in interaction data. Rankings generated by NetPert correlated better with wet-lab assays than rankings from betweenness centrality and related graph diffusion methods. The NetPert method provides useful, interpretable rankings of candidate drug targets in biological networks.

## Supporting information

S1 Fig Pearson correlation of log (fold-change) and NetPert weights with dissemination assay results. Dissemination assay results are from Ref. [21].
(TIF)

S1 Table Dissemination assay targets. Inhibitors used in the dissemination assay [21] with protein targets and reference source.
(TSV)

S2 Table AOD library results. Colony formation assay results for compounds from the Approved Oncology Drugs set IX library.
(TSV)

S3 Table EC library results. Colony formation assay results for compounds from the Epigenetic Compound library.
(TSV)

S4 Table Diffusion time analysis. Spearman correlation of NetPert, NetPert-Endpoints, and TieDIE rankings with dissemination assay results for varying diffusion times. Two-sided, single test p-values are provided in parentheses. Escaped density from the driver and density summed over response genes are also provided.
(TSV)

S5 Table Gene rankings and assay results. Mouse gene categories, Drug Repurposing Hub compounds, assay results, and rankings and scores from NetPert, betweenness centrality, and TieDIE. Gene categories are D: driver; R: response; DIR: intermediate having direct connections to the driver and at least one response gene; DI: intermediate connected directly to the driver but not to a response gene; IR: intermediate connected directly to a response gene but not the driver; DIIR: subset of DI and IR that are on a path of length 3 from driver to response genes; and I: intermediate directly connected to neither the driver nor a response gene. Dissemination and colony formation assay results are provided as a percentage of vehicle control, with 100 representing no effect and 0 representing complete inhibition. Compounds in the Drug Repurposing Hub with more than 5 targets are omitted.
(TSV)
