# Supplementary figures and images for: Prioritizing drug targets by perturbing biological network response functions
Source: PLoS Comput Biol. 2024 Jun 27;20(6):e1012195. doi: 10.1371/journal.pcbi.1012195 (PMC11236158; doi:10.1371/journal.pcbi.1012195)

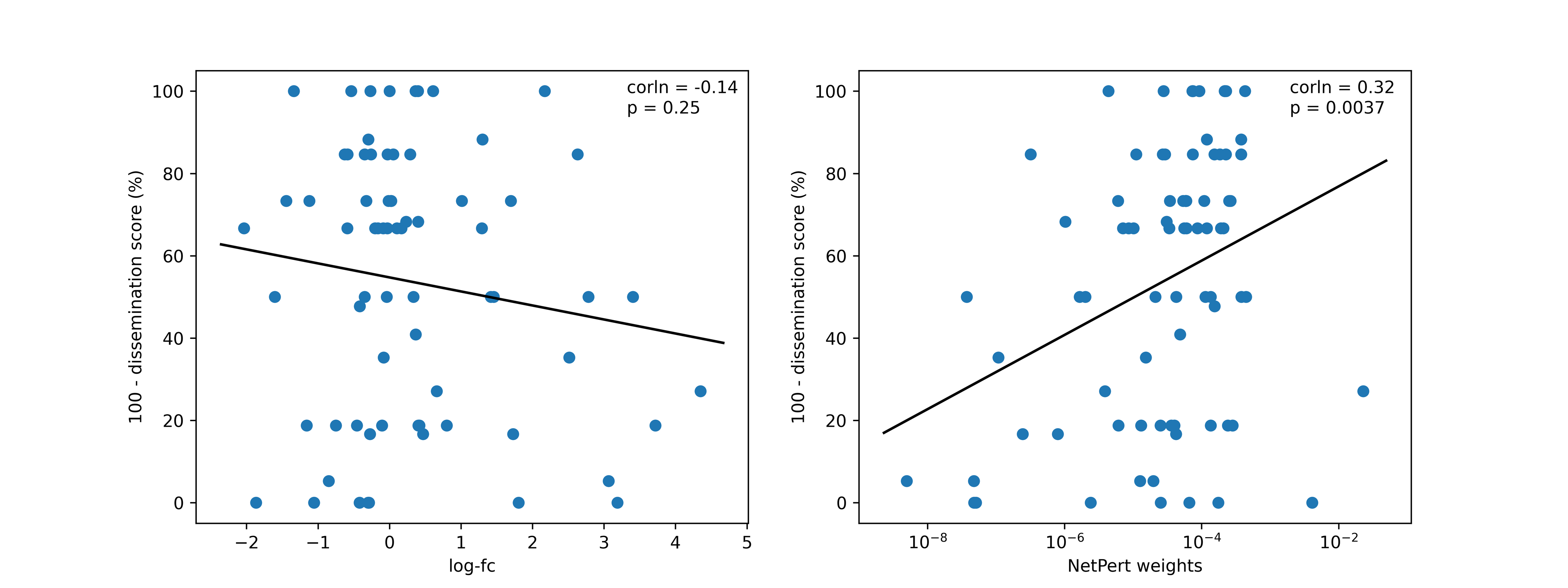

Supplement: S1 Fig — Dissemination assay results are from Ref. [21]. (TIF) [file pcbi.1012195.s001.tif]
